# C13 Megastigmane Derivatives From *Epipremnum pinnatum:* β-Damascenone Inhibits the Expression of Pro-Inflammatory Cytokines and Leukocyte Adhesion Molecules as Well as NF-κB Signaling

**DOI:** 10.3389/fphar.2019.01351

**Published:** 2019-11-28

**Authors:** San-Po Pan, Teresa Pirker, Olaf Kunert, Nadine Kretschmer, Scarlet Hummelbrunner, Simone L. Latkolik, Julia Rappai, Verena M. Dirsch, Valery Bochkov, Rudolf Bauer

**Affiliations:** ^1^Department of Pharmacognosy, Institute of Pharmaceutical Sciences, University of Graz, Graz, Austria; ^2^Department of Pharmaceutical Chemistry, Institute of Pharmaceutical Sciences, University of Graz, Graz, Austria; ^3^Department of Pharmacognosy, Molecular Targets, University of Vienna, Vienna, Austria

**Keywords:** β-damascenone, megastigmane, *Epipremnum pinnatum*, COX-2, IL-8, NF-κB, gene expression

## Abstract

In order to identify active constituents and to gain some information regarding their mode of action, extracts from leaves of *Epipremnum pinnatum* were tested for their ability to inhibit inflammatory gene expression in endothelial- and monocyte-like cells (HUVECtert and THP-1, respectively). Bioactivity-guided fractionation using expression of *PTGS2* (COX-2) mRNA as a readout resulted in the isolation of two C13 megastigmane glycosides, gusanlungionoside C (**1**) and citroside A (**3**), and the phenylalcohol glycoside phenylmethyl-2-*O*-(6-*O*-rhamnosyl)-ß-D-galactopyranoside (**2**). Further analysis identified six additional megastigmane glycosides and the aglycones β-damascenone (**10**), megastigmatrienone (**11**), 3-hydroxy-β-damascenone (**12**), and 3-oxo-7,8-dihydro-α-ionol (**13**). Pharmacological analysis demonstrated that **10** inhibits LPS-stimulated induction of mRNAs encoding for proinflammatory cytokines and leukocyte adhesion molecules, such as TNF-α, IL-1β, IL-8, COX-2, E-selectin, ICAM-1, and VCAM-1 in HUVECtert and THP-1 cells. **10** inhibited induction of inflammatory genes in HUVECtert and THP-1 cells treated with different agonists, such as TNF-α, IL-1β, and LPS. In addition to mRNA, also the upregulation of inflammatory proteins was inhibited by **10** as demonstrated by immune assays for cell surface E-selectin and secreted TNF-α. Finally, using a luciferase reporter construct, it was shown, that **10** inhibits NF-κB-dependent transcription. Therefore, we hypothesize that inhibition of NF-κB by β-damascenone (**10**) may represent one of the mechanisms underlying the *in vitro* anti-inflammatory activity of *Epipremnum pinnatum* extracts.

## Introduction

*Epipremnum pinnatum* (L.) Engl. (Araceae) is a slender to gigantic liana mostly distributed in tropical Southeast Asia and the Pacific Islands (Boyce, 1998; Wu et al., 2010). It is known as a popular indoor ornamental plant in western countries with the property to remove air pollutants, such as benzene, formaldehyde, and chloroform (Liu et al., 2011; Tada et al., 2010). In traditional Chinese medicine, aqueous leaf extracts have been used for detoxification and treatment of tendonitis, fractures, burns, carbuncles, sores, redness, and cancer (Chan et al., 2008; Siew et al., 2014). In other regions, such as Papua New Guinea and Rotuman, the plant has been used against inflammation, diabetes, and malaria (McClatchey, 1996; WHO, 2009). Even today, *Epipremnum* is part of herbal formulations, such as plasters, ointments, and medicinal wines (Ren, 2010; Li, 2013; Zhang 2013; Xiang and Zhang, 2014; Zong, 2014; Liu, 2015; Wang, 2015; Zong, 2015).

Previous investigations of lipophilic *n*-hexane leaf and stem extracts demonstrated a cytotoxic effect against T-47D breast cancer cells (EC_50_ = 2.90 µg/ml) by upregulation of c-myc and caspase 3 mRNA through the activation of PKC and tyrosine kinases pathways (Tan et al., 2005; Lan et al., 2007). Cytotoxic activity against P388 murine lymphocytic leukemia cells, MOLT4 human leukemia cells, KB human nasopharynx cancer cells, and SW 620 human colon adenocarcinoma cells was also reported from a diethylether extract (Lan et al., 2007). Antibacterial and antifungal activity was demonstrated by an aqueous extract (Sonawane et al., 2011), while the extracts obtained using supercritical CO_2_ inhibited pancreatic lipase (Hamzah et al., 2015). Moreover, the EtOH extract of aerial parts of *Epipremnum pinnatum* exerted anti-inflammatory activity in carrageenan-induced rat paw edema model, and induced analgesic effects in mice (Linnet et al., 2010).

Phytochemical screening of *Epipremnum pinnatum* revealed the presence of alkaloids, flavonoids, anthraquinones, tannins, glycosides, phenols, phytosterols, carbohydrates, and terpenoids (Arulpriya and Lalitha, 2011; Das et al., 2015; Meshram and Srivastava, 2015). One unstable cytotoxic compound has been isolated and identified as 5,6-dihyroxyindole. This compound rapidly undergoes autoxidation to form a black polymeric material (Wong et al., 1996; Goh, 1998).

The nuclear factor kappa B (NF-κB) family of transcription factors is ubiquitously expressed in mammalian cells (May and Ghosh, 1998) and is activated by complex signaling cascades (Hayden and Ghosh, 2012; Hoesel and Schmid, 2013). NF-κB is involved in the regulation of cell differentiation, proliferation, and cell survival. Moreover, it is a major player within the immune system (Pahl, 1999; Hoesel and Schmid, 2013).

NF-κB can be activated by pro-inflammatory stimuli including the cytokines lL-1β and TNF-α, as well as bacterial and viral antigens (Pahl, 1999; Hayden and Ghosh, 2012). Among the wide range of target genes controlled by NF-κB are genes encoding for cytokines like TNF-α, IL-1ß, cell adhesion molecules like ICAM-1, VCAM-1, and E-selectin or genes encoding for cyclooxygenase-2. All these proteins are essentially involved within the pathogenesis and regulation of inflammation. (Pahl, 1999; Liu et al., 2017).

The aim of the present study was to identify compounds, which are contributing to the anti-inflammatory activity of *E. pinnatum* and to evaluate their inhibitory activity on the inflammatory response in THP-1 and human umbilical vein endothelial cells to gain information about the underlying mechanisms.

## Materials and Methods

### General Experimental Procedures

UV spectra were recorded on a Thermo Scientific Ultimate 3000 diode-array detector. 1D and 2D NMR spectra were measured in methanol-d4 (δ_H_ 3.31/δ_C_ 49.0) either on a Varian UnityInova 600 spectrometer (^1^H: 400 MHz, ^13^C: 100 MHz) or on a Brucker Avance III NMR spectrometer (^1^H: 700.0 MHz, ^13^C: 166.0 MHz) equipped with a cryo probe. HR-MS was conducted on a Thermo Scientific Q Exactive Hybrid Quadrupole-Orbitrap equipped with electron spray ionization. ESI-MS were obtained on a LTQ XL mass detector in positive and in negative mode. Analytical HPLC was conducted on an Agilent 1100 with an Agilent 1100 diode-array detector. A RP-18 Kinetex (2.6 µm, 100 × 2.6 mm; Phenomenex) column was used for analytical separations employed with a 40-min gradient separation of 0.1% HCOOH in water-MeCN (95:5–0:100 over 30 min, 0:100 held for 10 min). Semipreparative HPLC was performed on a Shimadzu LC-20AT with a Shimadzu DPS-M20A detector on a LUNA RP-18 column (10 µm, 250 × 10 mm, Phenomenex). GC-MS was performed on an Agilent 7890A GC equipped with an Agilent 5975 VL MSD on a HP-5MS capillary column (30 m × 250 µm × 0.25 µm, Agilent). GC-MS method consisted of a 66-min temperature program with an initial temperature of 60°C held for 1 min followed by 4°C/min increase to 280°C held for 10 min. The flow rate of the carrier gas helium was 1.2 ml/min performed in splitless mode. The GC-MS system was operated in EI mode at 70 eV. The identification of the C13 aglycones was performed by comparison of measured chromatogram with available reference compounds or with library softwares (HPCH2205, NIST 08, Wiley138). Column chromatography was performed on a silica gel 60 (0.04–0.063 mm, Merck), and on a sephadex LH 20 (163 µm, GE-Healthcare). Separations were monitored using TLC 60 F_254_ (Merck) by staining with 5% sulfuric acid in EtOH and 1% vanilin in EtOH.

### Reference Compounds

α-Ionone (> 96%) **(14)**, β-ionone (96%) **(15)**, dihydro-β-ionone (90%) **(16)**, β-damascone (95%) **(17)**, and β-damascenone (> 98%) **(10)**, were purchased from Sigma-Aldrich. Megastigmatrienone/tabanone (mixtures of isomers) **(11)** was kindly provided by Lothar Streeck GmbH & Co. KG. Flavors & Fragrances (Germany, Hamburg).

### Plant Material

Leaves of *Epipremnum pinnatum* (L.) Engl. were collected in May 2014 from cultivated stock (IPEN ID number: XX-0-GZU-98100700) in the botanical garden of Graz, Graz, Austria. The identification was conducted by Christian Berg (Institute of Plant Sciences, University of Graz, Austria). Voucher specimen (080514_EPI_Fol_tot_GZU, 080514_EPI_Fol_pulv_GZU) has been deposited at the herbarium of the Department of Pharmacognosy, University of Graz, Austria. The fresh plant material was air-dried for 3 weeks at 30°C in an air-flow chamber.

### Extraction and Isolation

Dried leaves (47 g) were crushed in a blender and submitted to ASE (Dionex Accelerated Solvent Extractor-200) for successive extraction using *n*-hexane, dichloromethane, and MeOH as solvents. ASE conditions were set as follows: pressure, 69 bar; temperature, *n*-hexane: 72°C; dichloromethane: 44°C; MeOH: 98°C; static, 5 min; heat, 5 min; flush, 40%; purge 60 s; cycles, 3; total solvent volume of each solvents: 33 ml; amount of samples: 10.0 g each cell. The MeOH extract (5.59 g) was dried under N_2_ and chromatographed on a Silica gel column with mixtures of *n*-hexane-EtOAc-MeOH gradient system (from 100:0:0 to 0:0:100) as eluents. Fractions with similar compositions were combined according to TLC monitoring, producing fractions E1 to E20. Fraction E16 (758 mg), obtained with *n*-hexane-EtOAc-MeOH (1:1:2) was further separated on a Sephadex LH 20 column using MeOH as eluent. The collected volume of each fractions was 20 ml. Fractions were combined to 11 fractions after TLC monitoring (S1–S11). Fractions S3-S6 were subjected to semi-preparative HPLC using a RP-18 column (Phenomenex LUNA^®^ 10 µm, 250 × 10 mm) as stationary phase and a gradient system of MeCN-water (15:85–100:0) as mobile phase (flow rate, 4.0 ml/min) over 30 min at 30°C column temperature to afford **1** (2.56 mg), and a mixture of **2** and **3** (1.93 mg). Other C13 compounds and aglycones were identified using GC-MS and HR-MS in fractions S3-S6 by comparison with available reference compounds or literature data as specified in the text.

*Gusanlungionoside C* (**1**). Colorless powder; λ_max_ 245.0 nm; ^1^H NMR and ^13^C NMR data, see **Supplementary Data**; HRESIMS *m/z* 519.2808 [M + H]^+^ (calc for C_25_H_43_O_11_, 519.2808); ESIMS positive mode *m/z* 519 [M + H]^+^, 373 [M + H–Rha]^+^, 211 [M + H–Rha–Glc]^+^; ESIMS negative mode *m/z* 563 [M–H + HCOOH]^+^, 517 [M–H]^−^, 371 [M–H–Rha]^−^, 161 [M–H–356]^−^.

*Phenylmethyl-2-O-(6-O-rhamnosyl)-ß--galactopyranoside* (**2**). Colorless powder; λ_max_ 247.0 nm; ^1^H NMR and ^13^C NMR data, see **Supplementary Data**; HRESIMS *m/z* 417.1763 [M + H]^+^ (calc for C_19_H_29_O_10_, 417.1763); ESIMS negative mode *m/z* 461 [M–H+ HCOOH]^+^, 415 [M–H]^−^, 269 [M–H–Rha]; 161 [M–H–254]^−^.

*Citroside A* (**3**). Colorless powder; λ_max_ 234.0 nm; ^1^H NMR and ^13^C NMR data, see **Supplementary Data**; HRESIMS *m/z* 387.2020 [M + H]^+^ (cald for C_19_H_31_O_8_, 387.2020); ESIMS in negative mode *m/z* 431 [M–H + HCOOH]^+^, 385 [M–H]^−^, 223 [M–H–Glc]^−^, 138 [M–H–247]^−^.

### qPCR Analysis of COX-2 mRNA Level

The human monocytic cell line THP-1 was obtained from the European Collection of Cell Cultures (ECACC, catalogue No: 88081201) and maintained in medium RPMI 1640 (Gibco, ThermoFisher Scientific Inc., NY, USA) containing 2 mM L-glutamine, 10 mM HEPES (Gibco), 10% fetal bovine serum (FBS, Gibco), 100 units/ml penicillin (Gibco) and 100 µg/ml streptomycin (Gibco) at 5% CO_2_ and 37°C in humidified atmosphere. To initiate the monocyte to macrophage differentiation, THP-1 were seeded 1 × 10^6^/ml in a 24-well plate and incubated with medium containing 12 nM phorbol-12-myristate-13-acetate (PMA, Sigma-Aldrich) for 48 h. Cells were further incubated with test compounds for 1 h followed by the stimulation with 7.5 ng/ml LPS for additional 3 h. Subsequently, total RNA was extracted with the GenElute^™^ mammalian total RNA miniprep kit (Sigma-Aldrich) and reverse transcribed with the high-capacity cDNA reverse transcription kit (Applied Biosystems^®^) according to manufacturer's instructions. The cycler conditions was set as followed: 25°C for 10 min, 37°C for 120 min, and 85°C for 5 s. Quantitative real-time PCR (qPCR) was performed on a ABI 7300 real-time PCR Systems (Applied Biosystems^®^) with primers designed with Primer Express Software (Applied Biosystems^®^). The sequences for COX-2 were as follows: COX-2 forward: 5′-GAA-TCA-TTC-ACC-AGG-CAA-ATT-G-3′, COX-2 reverse: 5′-TCT-GTA-CTG-CGG-GTG-GAA-CA-3′, and COX-2 probe: 5′-FAM-TCC-TAC-CAC-CAG-CAA-CCC-TGC-CA-TAMRA-3′. TaqMan probe was used against endogenous control GAPDH (pre-developed TaqMan^®^ assay, Applied Biosystems^®^). The mRNA expression was quantified using the ΔΔCt method. The instrument was set to 50°C for 2 min, 95°C for 10 min, followed by 40 PCR cycles of 95°C for 15 s and 60°C for 1 min (Lajter et al., 2015).

### IL-8 ELISA

Human immortalized umbilical vein endothelial cells (HUVECtert) were seeded at a density of 4000 cells/well in a 96-well plate with M199 medium supplemented with 20% FCS, antibiotics (100 units/ml penicillin, 100 µg/ml streptomycine, 250 ng/ml fungizone, Lonza), 2 mM L-glutamine, and 12 µg/ml endothelial cell growth supplement/90 µg/ml heparin (PromoCell). After 24 h, the medium was replaced with medium containing 3% FCS and tested compounds. Treatment was performed in quintuplicates. After 15 min of preincubation with inhibitors the cells were stimulated with 0.3 ng/ml TNF-α or 30 ng/ml LPS (end concentration) and incubated for 6 h. Subsequently, medium supernatant was used to quantify IL-8 protein concentration with DuoSet ELISA development kit (R&D Systems) (Oskolkova et al., 2017).

### Analysis of NF-κB-dependent Genes

Analysis of NF-κB-dependent genes was performed in human umbilical vein endothelial cells (HUVECtert) and the human monocytic cell line THP-1. HUVECtert cells were seeded in a 12-well plate with M199 medium supplemented with 20% FCS, antibiotics (100 units/ml penicillin, 100 µg/ml streptomycine, 250 ng/ml fungizone; Lonza), 2 mM L-glutamine, and 12 µg/ml endothelial cell growth supplement/90 µg/ml heparin (PromoCell). Cells were grown to full confluence, before they were used for experiments. THP-1 cells were cultured as described above, seeded at density of 1x10^6^ cells/well in a 12-well plate, and used for experiments without inducing differentiation into macrophages by phorbol ester treatment. For experiment, growing medium was replaced with medium containing 3% FCS and 20 mM HEPES. Cells were pre-incubated with sample compounds for 30 min. Afterward, LPS (30 ng/ml), TNF-α (0.3 ng/ml), or IL-1β (1 ng/ml) were added and incubated for 4 h at 37°C. Subsequently, cells were lysed in RNAzol (Molecular Research Center Inc.) and total RNA was isolated according to manufacturer's instructions.

900 ng RNA were reversely transcribed using murine leukemia virus reverse transcriptase (MuLV from Applied Biosystems^®^) and oligo (dT)_16_ primers (Invitrogen). T100^™^ Thermal Cycler was set to following conditions: 25°C for 10 min, 42°C for 45 min, and 95°C for 5 min. Quantitative real time PCR was performed on a StepOnePlus instrument (Applied Biosystems^®^) using SYBRGreen master mix (PCR Biosystems Ltd). Primer sequences are presented in **Table 1**. mRNA expression was quantified using the ΔΔCt method. Amplification program was set as following: 30 s at 90°C, followed by 40 PCR cycles of 3 s at 95°C and 30 s at 60°C; melting point analysis in 0.1°C steps. The mRNA level of each target gene was normalized to expression of β2-microglobulin. Fold expression was defined as fold increase relative to β2-microglobulin-normalized level of expression in mock-stimulated treatment with vehicle) cells (0.1% DMSO).

**Table 1:** List of primer pairs used for SYBRGreen RT-qPCR.

Primer	Sequence forward	Sequence reverse
VCAM-1	5´-AGA GCA CGA GAA GCT CAG GA-3´	5´-GCG GAG ACA GGA GAC ACA GT-3´
ICAM-1	5’- AGC TTC TCC TGC TCT GCA AC-3’	5´-TGG GAA AGT GCC ATC CTT TA- 3´
E-selectin	5’-GGT TTG GTG AGG TGT GCT C-3'	5’-TGA TCT TTC CCG GAA CTG C-3’
TNF-α	5’-GGC TCC AGG CGG TGC TTG TTC-3’	5’-AGA CGG CGA TGC GGC TGA TG-3’
IL-1β	5’-GGA GAA TGA CCT GAG CAC CT-3’	5’-AGT TCA TAT GGA CCA GAC AT-3’
β2-microglobulin	5′-ATT CAC CCC CAC TGA GAC TG-3′	5′-TGC TAT TTC TTT CTG CGT GC-3′

### TNF-α ELISA

THP-1 cells were seeded at a density of 1x10^5^ cells per well in a 96-well plate with RPMI 1640 supplemented with 20 mM HEPES, 3% FCS and 100 units/ml penicillin (Gibco) and 100 µg/ml streptomycin. After 30 min pre-incubation with test compounds, cells were stimulated with 30 ng/ml LPS and incubated for another 4 h. Subsequently, TNF-α protein was quantified in medium supernatant by a DuoSet ELISA kit (R&D Systems).

### Cell-based E-selectin ELISA

HUVECtert cells were seeded at density of 4000 cells per well in 96 well plates and incubated for 48 h before experiment. Afterward, medium was replaced with medium containing 3% FCS and 20 mM HEPES. Cells were pretreated with sample compounds for 30 min, followed by stimulation with LPS (30 ng/ml) for 4 h. Cells were fixed with 0.1% glutaraldehyde in PBS for 15 min at 4°C. Blocking was performed with 5% BSA in PBS for 1 h at 37°C. Afterward, cell-bound E-selectin was analyzed using a DuoSet ELISA development kit (R&D Systems).

### NF-κB-Driven Luciferase Reporter Gene Transactivation

The stable cell line HEK 293/NF-κB-luc was obtained from Panomics (catalog RC0014). TNF-α stimulation of these cells induces the expression of the luciferase reporter gene that is regulated by multiple copies of NF-κB response elements. For the assay, cells were seeded in 96 well plates at a density of 4 × 10^4^ cells per well in serum-free DMEM overnight. Cells were stained with 2 µM cell tracker green (CTG, Thermo Scientific) for 1 h, followed by subsequent treatment with respective compounds for 1 h and then stimulation with 2 ng/ml TNF-α for 4 h, which initiated NF-κB activation. Fluorescence of CTG-stained cells and luminescence of the luciferase product were measured on a Tecan SPARK^®^. The obtained luminescence values were normalized to CTG-derived fluorescence related to DMSO control. Normalized fluorescence units were used as indicator for cell number (Fakhrudin et al., 2014). To test whether β-damascenone inhibits NF-κB signaling *via* an electrophilic attack, 5 mM glutathione was added shortly before test compounds to all samples.

### Cell Viability Assay

100.000 THP-1 cells/ml (100 µl/well) were seeded in 96-well plates and incubated with 12 ng/ml PMA for 48 h. HUVECtert cells were seeded at density of 7,000 cells/well in 96 well plates and incubated for 48 h. Afterward, medium was replaced to medium containing 3% FCS and indicated concentrations of sample compounds. After 24 h of incubation, medium was exchanged to medium with 0.2 mg/ml 2,3-bis-(2-methoxy-4-nitro-5-sulfophenyl)-2H-tetrazolium-5-carboxanilide (Molecular Probes) and 5 nM phenazine methosulfate (Acros-Organic). After 1.5 h of incubation, 1 µg/ml Hoechst-33342 (final concentration) was added. Cells were incubated for 30 min. Afterward, absorbance at 450 nm (XTT), number of stained nuclei (Hoechst) and confluence were measured using the EnSight multimode plate reader equipped with Kaleido data acquisition and analysis software (PerkinElmer).

### Statistical Analysis

Statistical analysis was calculated by one-way analysis of variance (ANOVA) with Bonferroni *post hoc* test using IBM SPSS Statistics 25 software. In graphs, data were expressed as mean values ± standard deviation (SD). Statistical significance was expressed as p values: p > 0.05 *, p > 0.01 **, p > 0.001 ***.

## Results and Discussion

**Figure 1:**
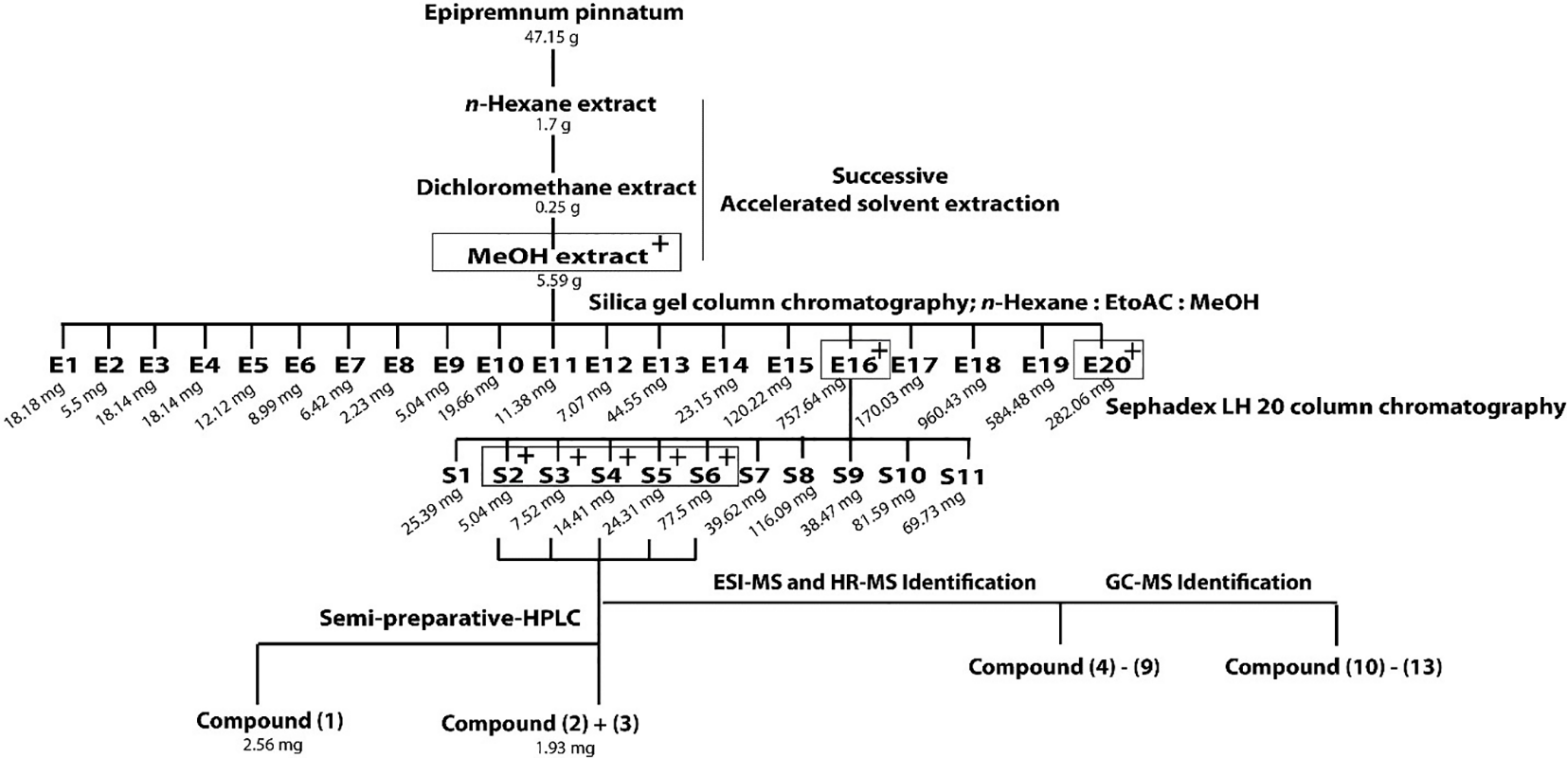
Extraction and isolation scheme of anti-inflammatory components from leaves of *Epipremnum pinnatum*. The plant material was successively extracted with *n*-hexane, dichloromethane and methanol. The active methanol extract was further fractionated on a silica gel column to gain E1-E20. Fraction E16 was fractionated on a Sephadex LH20 column to produce fractions S1-S11. The most active fraction (S2-S6) were combined to isolate **1** and a mixture of **2** and **3**. Compounds **4**-**9** and **10**-**13** could be identified with ESI-MS, HR-MS and GC-MS. Active fractions are determined using qPCR analysis of COX-2 mRNA and are indicated with "^+"^ in grey boxes.

Out of three successively prepared extracts (*n*-hexane, dichloromethane, and MeOH) from *E. pinnatum* leaves, the MeOH extract inhibited the LPS-induced COX-2 mRNA expression most potently (inhibition at 20 µg/ml: *n*-hexane: 10.8 ± 10.4%, DCM: no effect, MeOH: 54.3 ± 9.2%) and was, therefore, subjected to activity-guided isolation of the active compounds. First, the MeOH extract was fractionated by silica gel column chromatography using mixtures of *n*-hexane, ethyl acetate, and water. After TLC comparison and combining similar fractions (175 fractions in total), 20 fractions E1 to E20 were obtained. Subsequent pharmacological testing revealed two active fractions, E16 and E20 (inhibition of LPS-induced COX-2 mRNA expression at 20 µg/ml: E16: 32.7 ± 6.7%, E20: 31.3 ± 7.1%). E16 was further separated on a size exclusion column using Sephadex LH 20 to gain 11 fractions (S1–S11). Active fractions (S3–S6) were combined (inhibition at 20 µg/ml from 20.9 to 71.8%) and compounds of interest were separated on a RP18 column by semi-preparative HPLC to yield gusanlungionoside C **(1)** and a mixture of phenylmethyl-2-*O*-(6-*O*-rhamnosyl)-ß-D-galactopyranoside **(2)** and citroside A **(3)**. **Figure 1** depicts the isolation scheme. Structure elucidation was carried out using 1-D proton NMR, 2-D NMR (DQFCOSY, HSQC, HMBC), ESI-MS, HR-MS, and GC-MS experiments. For NMR spectra and data, see **Supplementary Data**.

**Figure 2:**
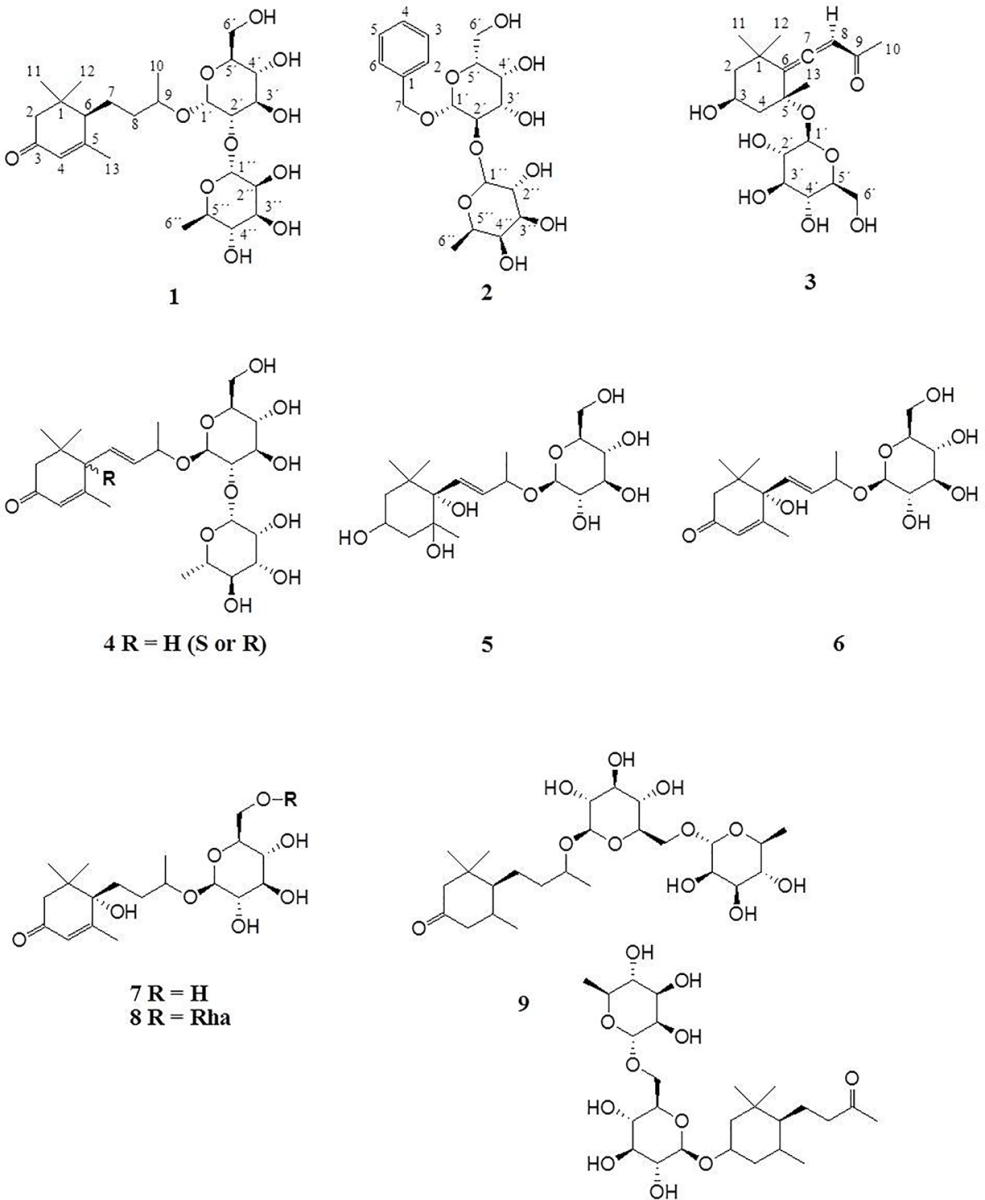
Megastigmane glycosides **1**–**9**.

Compound **1** was isolated as colorless powder. HR-MS and NMR spectroscopic data indicated a megastigmane derivative with a trimethylcyclohexane moiety attached to a hexose and a deoxyhexose unit. HR-MS suggested a molecular formula of C_25_H_42_O_11_, according to a monoisotopic mass at m/z 519.2808 [M + H]^+^ (calculated for C_25_H_43_O_11_, 519.2808). ESI-MS detected a deprotonated molecule at *m/z* 563 [M–H + HCOOH]^+^, which fragmented to its molecular ion at *m/z* 517 [M–H]^−^. This compound was subsequently fragmented, losing a rhamnose molecule (*m/z* 371, [M–H–Rha]^−^) and its aglycon, with a remaining glucose molecule at *m/z* 161 [M–H–Rha – 210]^−^. The constitution of **1** was determined by complete assignment of 1H, DQF-COSY, HSQC, and HMBC spectra, which indicated the presence of a megastigmane derivative with two sugar units. The homonuclear coupling constant (H-1′, 7.8 Hz) of the glucose and the carbon chemical shift (69.5 ppm) of C-5 of rhamnose indicated the presence of a *β*-glucopyranose and a *α*-rhamnopyranose. HMBC correlation between the anomeric proton (H-1') of the glucose and the carbon at 75 ppm indicated that the glucose was attached to C-9 of the aglycon, while HMBC correlation between the anomeric proton of the rhamnose (H-1′′) and the carbon at 78.3 ppm pointed to the attachment of this sugar to C-2′ of the glucose. Hence, the constitution of the compound was determined as depicted in **Figure 2**. Three DBEs are provided by the ring fusions, two by the double bonds in the aglycon. The relative configurations of the stereogenic centers C-6 and C-9 of the aglycon were elucidated by comparison of the proton and carbon resonance values with literature data. The values fit well with compound 3 of the publication by Yu et al. (2011) with reported absolute configurations 6*S*, 9*R*, assigned to a compound named gusanlungionoside C.

A mixture of **2** and **3** was isolated as colorless powder. According to proton NMR, the sample comprised two major glycosidic compounds. As the aglycon parts of these components were chemically quite different, complete resonance assignments for the non-sugar portions of the compounds were possible. HR-MS measurement identified protonated molecules at *m/z* 417.1764 (**2**) and 387.2020 (**3**) [M + H]^+^ (calc for C_19_H_29_O_10_, 417.1764; calc for C_19_H_31_O_8_, 387.2020). ESI-MS of **2** showed characteristic fragment loss of a formic acid adduct at *m/z* 461 [M–H + HCOOH]^+^, with further loss at *m/z* 415 [M–H]^−^, 269 [M–H–Rha]^−^, and 161 [M–H–Rha – 108]^−^, indicating a cleavage of rhamnose and its aglycon with a remaining glucose molecule. This is in accordance with the NMR data. According to the observed resonances in proton NMR and HSQC, the disaccharide was formed by a hexopyranose in the β-form and a β-rhamnopyranose. The correlations in the HMBC spectrum between H-7 (4.94 ppm) and C-1' (101.6 ppm), and H-1' (4.42 ppm) and C-7 (71.5 ppm), respectively, indicated that the benzyl alcohol was attached to the anomeric carbon of the hexose. The three bond correlation between H-1'' (5.20 ppm) and 79.2 ppm indicated that the rhamnose was attached to C-2′. Due to severe signal overlap in the NMR spectra of the hexoses of **2** and **3**, it was not possible to determine the relative configuration at C-4′, i.e. it was not possible to determine whether the β-hexose is a glucose or galactose. However, the relatively low carbon shift value of C-2' (79 ppm) points more to a galactose, because for glucose more than 82 ppm would be expected. Compound **2** has therefore been tentatively assigned as phenylmethyl-2-*O*-(6-*O*-rhamnosyl)-ß-D-galactopyranoside.

The MS of compound **3** exhibited a formate adduct at *m/z* 431 [M–H + HCOOH]^+^, with sequential fragment loss to its molecular ion at *m/z* 385 [M–H]^−^, 223 [M–H–Glc]^−^, and 138. According to NMR analysis, compound **3** was a C13 megastigmane derivative with a trimethylcyclohexane portion attached to a hexose. Complete analysis of HMBC correlations revealed that the aglycon was the so-called “grasshopper ketone”with a β*-*glucopyranose moiety attached to C-5. This compound is a cumulene with two different terminal substituents (H, acyl moiety) at C-8. This structural feature leads to a chiral axis and due to the stereogenic centers C-3 and C-5, respectively, to two possible diastereomers with different spatial orientation of the terminal groups, which are described as citroside A and citroside B in the literature. The NMR-resonance values of component A fit much better with those of citroside A (Umehara et al., 1988; Zhang et al., 2010).

### HR-MS and ESI-MS Identification of Megastigmane Glycosides

ESI-MS and HR-MS analyses of the initial combined fractions S3-S6 showed six additional compounds with characteristic fragmentations of megastigmane glycosides. Since the megastigmane aglycones can vary in the degrees of saturation and hydroxylation (Gribble, 1991; Hou et al., 2016), a structural determination could be made in comparison to the isolated compounds **1** and **3**. **Table 2** depicts the mass fragmentation of identified compounds. They were identified as gusanlungionoside A or B (**4**), actinidioionoside (**5**), roseoside (**6**), 7,8-dihydroroseoside (**7**), blumenol B 9-*O*-α-L-rhamnopyranosyl-(1 6)-β-D-glucopyranoside (**8**), sedumoside J or G (**9**), as described below.

**Table 2:** ESI-MS and HR-MS analysis of initial fraction S3-S6 – megastigmane glycosides.

Compounds	RT [min]	UV [nm]	Negative mode (ESI-MS)		Positive mode (HR-MS)		MW	MW aglycon	Reference
			Negative ion [m/z]	Fragment ions [m/z]	Positive ion [m/z]	molecular formula [M + H]+			
Isolated compounds									
**1**	9.03	240	[M-H+HCOOH]^+^: 563	517, 371, 161	[M+H]^+^: 519.2808	C_25_H_43_O_11_	518	210	(Yu et al., 2011)
**2**	6.61	247	[M-H+HCOOH]^+^: 461	415, 269, 161	[M+H]^+^: 417.1763	C_19_H_29_O_10_	416	108	–
**3**	6.79	234	[M-H+HCOOH]^+^: 431	385, 223, 153, 138	[M+H]^+^: 387.2020	C_19_H_31_O_9_	386	224	–
Identified compounds									
**4**	8.80	240	[M-H+HCOOH]^+^: 561	315, 325, 306, 246	[M+H]^+^: 517.2654	C_25_H_41_O_11_	516	208	(Yu et al., 2011)
**5**	5.92	196, 222	[M-H+HCOOH]^+^: 451	405, 243, 167, 149	[M+H]^+^: 407.2285	C_19_H_35_O_9_	406	244	(Otsuka et al., 2003)
**6**	7.42	247	[M-H+HCOOH]^+^: 431	385, 223, 153, 138	[M+H]^+^: 387.2021	C_19_H_31_O_9_	386	224	(Herderich et al., 1992; Otsuka et al., 1995; Shen and Terazawa, 2001)
**7**	6.65	243	[M-H+HCOOH]^+^: 433	387, 225	[M+H]^+^: 389.2177	C_19_H_33_O_9_	388	226	(Shen and Terazawa, 2001)
**8**	7.23	240	[M-H+HCOOH]^+^: 579	533, 387, 225	[M+H]^+^: 535.2762	C_25_H_43_O_12_	534	226	(Tommasi et al., 1996)
**9**	8.71	244	[M-H+HCOOH]^+^: 565	519, 373, 161	[M+H]^+^: 521.2969	C_25_H_45_O_11_	520	212	(Osamu et al., 2009; Morikawa et al., 2007)

In the HR-MS spectra, **4** showed a protonated molecule at 517.2654 [M + H]^+^ with a calculated formula of C_25_H_41_O_11_. ESI-MS fragmentation exhibited a formate adduct ion at *m/z* 561 [M–H + HCOOH]^+^, with the sequential fragments at *m/z* 517 [M–H]^−^, 371 [M–H–Rha]^−^, 161 [M–H–Rha–210]^−^. Compared to **1**, compound **4** differed by *m/z* 2, indicating a structural difference by a double bond. These measured values fit well to the stereochemical isomers assigned as gusanlungionosides A and B described by Yu et al. (Yu et al., 2011).

Compound **5** showed a protonated molecular ion at *m/z* 407.2285 [M + H]^+^ corresponding to the formula C_19_H_35_O_9_. ESI-MS revealed a formate adduct ion at *m/z* 451 [M–H + HCOOH]^+^. Its molecular ion at *m/z* 405 [M–H]^−^ fragmented in *m/z* 243 [M–H–Glc]^−^, 167 [M–H–Glc–76]^−^, 149 [M–H–Glc–94]^−^. The aglycon with the deprotonated molecule at *m/z* 243 differed from the aglycon of **1** by additional *m/z* 34 indicating a difference of a saturated double bond and two oxygen moieties. This compound was described as actinidioionoside by Otsuka et al. (2003).

Compound **6** shares the same fragmentation pattern and molecular formula as **3**. HR-MS detected a protonated molecule at *m/z* 387.2021 [M + H]^+^ (calc C_19_H_31_O_9_). This compound has been assigned as roseoside according to literature values (Herderich et al., 1992; Otsuka et al., 1995; Shen and Terazawa, 2001).

In HR-MS, **7** exhibited a monoisotopic mass at *m/z* 389.2177 [M + H]^+^ (calc C_19_H_33_O_9_). ESI-MS displayed a formate adduct ion at *m/z* 433 [M–H + HCOOH]^+^, with further fragments at *m/z* 387, and 225. Compared to **6**, compound **7** varied by additional *m/z* 2 indicating a saturated double bond in its structure. 7,8-dihydroroseoside fits well to the measured and described literature values (Shen and Terazawa, 2001).

Compound **8** is a megastigmane diglucoside comprised of **7** additionally linked with a rhamnose. HR-MS exhibited a protonated molecule at *m/z* 535.2762 [M + H]^+^, indicating a molecular formula of C_25_H_43_O_12_. ESI-MS showed characteristic fragments at *m/z* 579 [M–H + HCOOH]^+^, 533 [M–H]^−^, 387 [M–H–Rha]^−^, and 225 [M–H–Rha–Glc]^−^. This compound has been previously isolated by Tommasi et al. (1996) and assigned as blumenol B 9-*O*-α--rhamnopyranosyl-(1 6)-β--glucopyranoside.

Compound **9** gained a molecular ion peak at *m/z* 521.2969 [M + H]^+^ (calc C_25_H_45_O_11_). ESI-MS fragmentation showed deprotonated fragment ions at *m/z* 565 [M–H + HCOOH]^+^, 519 [M–H]^−^, 373 [M–H–Rha]^−^, and 161 [M–H–Rha–212]^−^. These results differed from **1** by additional *m/z* 2, indicating a saturation of one double bond. Two compounds fitting to the measured values are sedumoside J or sedumoside G (Morikawa et al., 2007; Osamu et al., 2009). The position of the sugar linkage could not be determined by mass spectrometry.

**Figure 2** depicts the identified megastigmane glycosides **1**–**9**.

### GC-MS Identification of Megastigmane Aglycones

C13 megastigmane derivatives represent a large group of natural products important as aromatic components in fruits and plants. In order to identify the low molecular weight volatile megastigmane aglycones, capillary GC-MS has been employed. GC-MS of the fractions S3 to S6, as shown in **Figure S5** (supporting information), allowed the identification of the four megastigmane aglycones β-damascenone **(10)**, megastigmatrienone **(11)**, 3-hydroxy-β-damascone (**12**), and 3-oxo-7,8-dihydro-α-ionol (**13**). The identification was performed by comparing the MS fragmentation patterns with library data (HPC2205, NIST08, Wiley138) and reference compounds. The mass spectra are dispicted in the supporting information. The other peaks were not identified because they were from a fraction, which did not show activity.

### *In Vitro* Pharmacology of Megastigmane Derivatives

**Figure 3:**
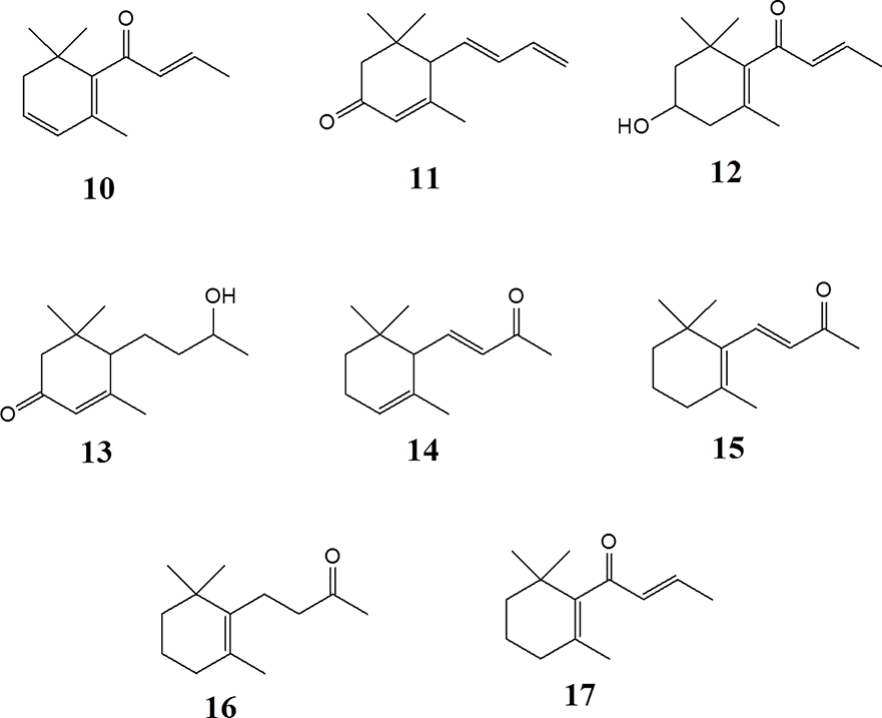
Megastigmane aglycones **10**-**17**.

**Figure 4:**
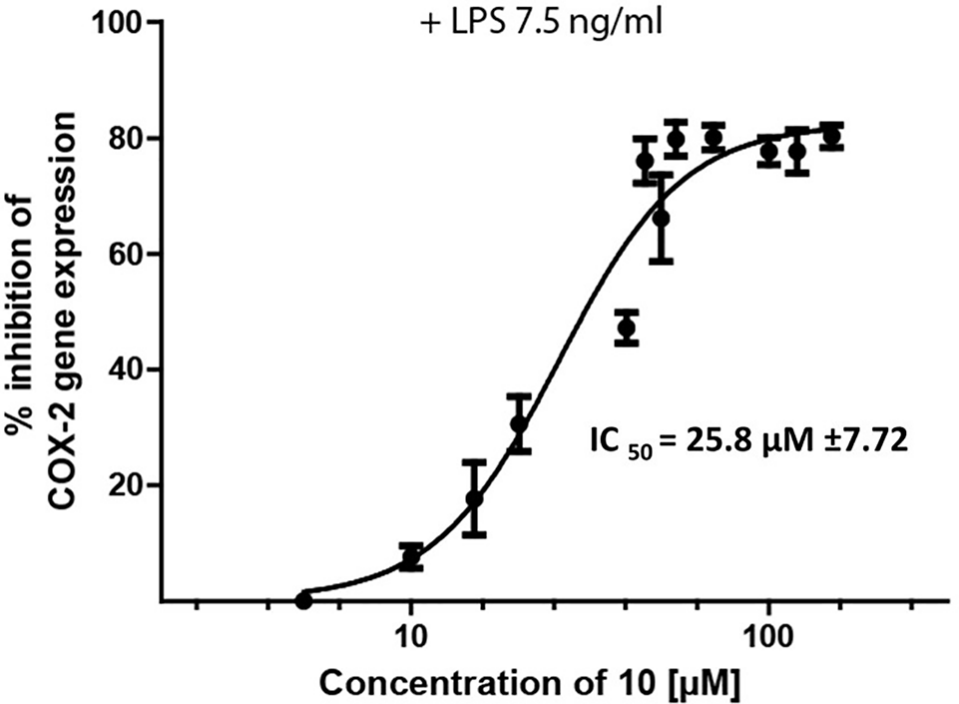
Inhibition of LPS-induced (7.5 ng/ml) *PTSG2* (COX-2) mRNA expression by **10**. Cells were pretreated with β-damascenone (**10**) for 1 h followed by LPS stimulation for 3 h. Total RNA was isolated and reverse transcribed to cDNA. cDNA was amplified and expression of *PTSG2* (COX-2) was normalized to the expression of GAPDH mRNA. Data are presented as mean ± SE (n = 3).

*Epipremnum pinnatum* is used in traditional medicine for treatment of various inflammation-associated conditions. We addressed the question whether megastigmane derivatives identified in this project can inhibit inflammatory reactions in cell types relevant to inflammation, such as endothelial cells and mononuclear leukocytes. To this end, LPS-induced upregulation of the pro-inflammatory *PTGS2* (COX-2) mRNA in the monocyte-like THP-1 cell line was used as a readout. In addition to the isolated compounds, α-ionone (**14**), β-ionone (**15**), 7,8-dihydro-β-ionone (**16**), and damascone (**17**), which all have similar structures, were available as reference compounds. **Figure 3** depicts the megastigmane aglycones **10–17**. Analysis of the relative inhibitory activity showed that out of the tested compounds, only compound **10** was able to inhibit *PTGS2* (COX-2) mRNA expression (**Table 3**). The inhibitory effect was dose-dependent (**Figure 4**). Since only **10** demonstrated significant activity, further studies were focused on the pharmacological analysis of this substance.

**Table 3:** IC50 values of isolated and reference compounds in COX-2 gene expression assay in THP-1 macrophages. n.a. no activity was observed up to a concentration of ^a^ 20 µg/ml; ^b^ 50 µM.

Compound	IC50 ± SD COX-2 mRNA
**1**	n.a.^a^
**2** and **3**	n.a.^a^
**10**	25.8 ± 7.7 µM
**11**	n.a.^b^
**14**	n.a.^b^
**15**	n.a.^b^
**16**	n.a.^b^
**17**	n.a.^b^
Dexamethasone	3.3 ± 1.2 nM
Parthenolide	6.0 ± 1.7 µM
BAY-11-7082	7.3 ± 1.8 µM

**Figure 5:**
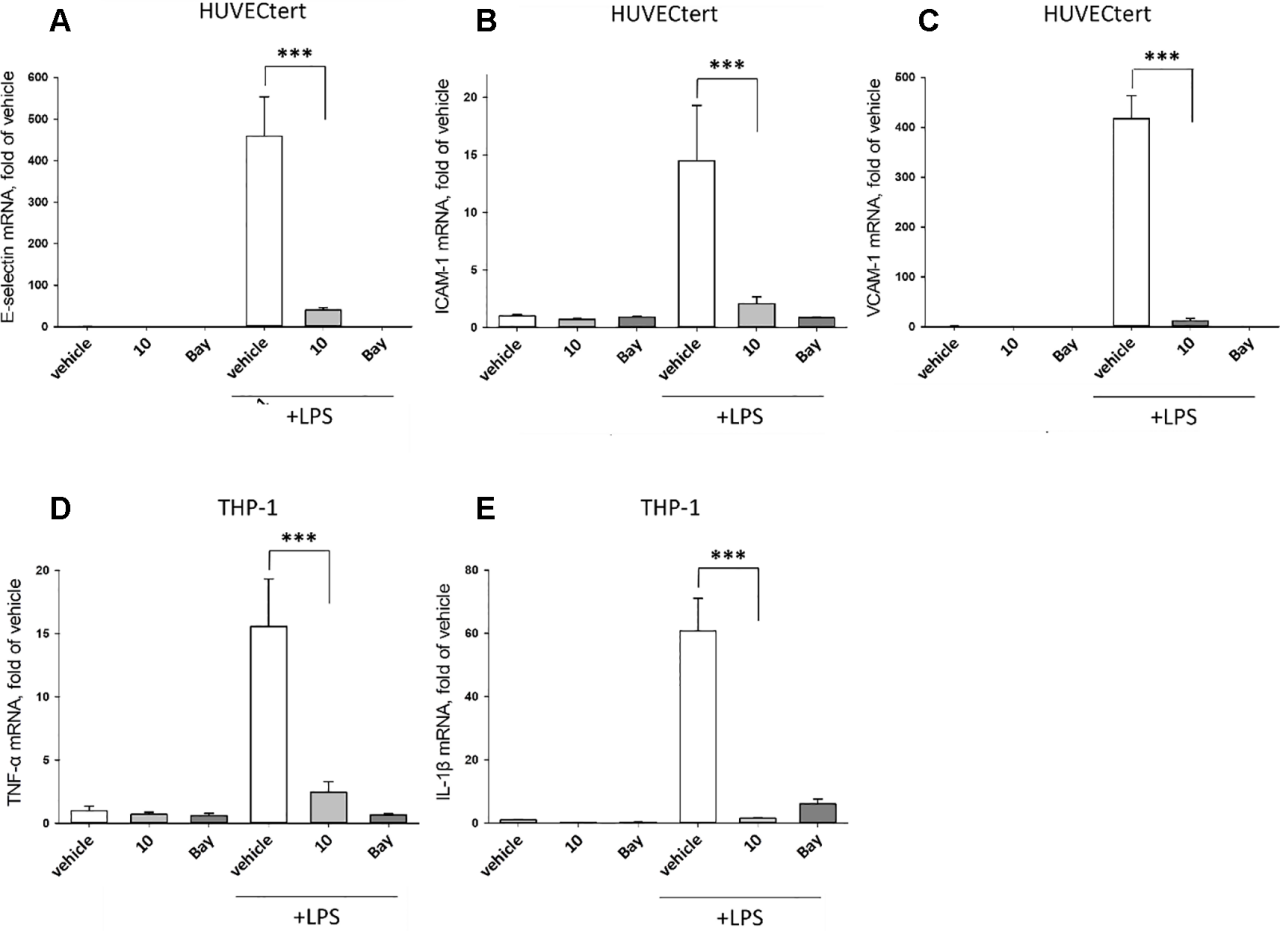
Inhibition of **(A)** E-selectin, **(B)** ICAM-1 and **(C)** VCAM-1 gene expression in HUCECtert cells, and inhibition of **(D)** TNF-α and **(E)** IL-1β gene expression in THP-1cells stimulated with LPS. Cells were pretreated with 30 µM β-damascenone (**10**) for 30 min followed by stimulation with LPS (30 ng/ml) for 4 hours. Basal values refer to vehicle-stimulated cells. Bay-11–7082 (5 µM) served as a positive control. Isolation of total RNA, cDNA synthesis and real-time PCR were performed as described in materials and methods section. Results are normalized to β2-microglobulin. Data are presented as mean ± SE (n=4). P-values are shown as *< 0.5, **<0.01 ***<0.001.

**Figure 6:**
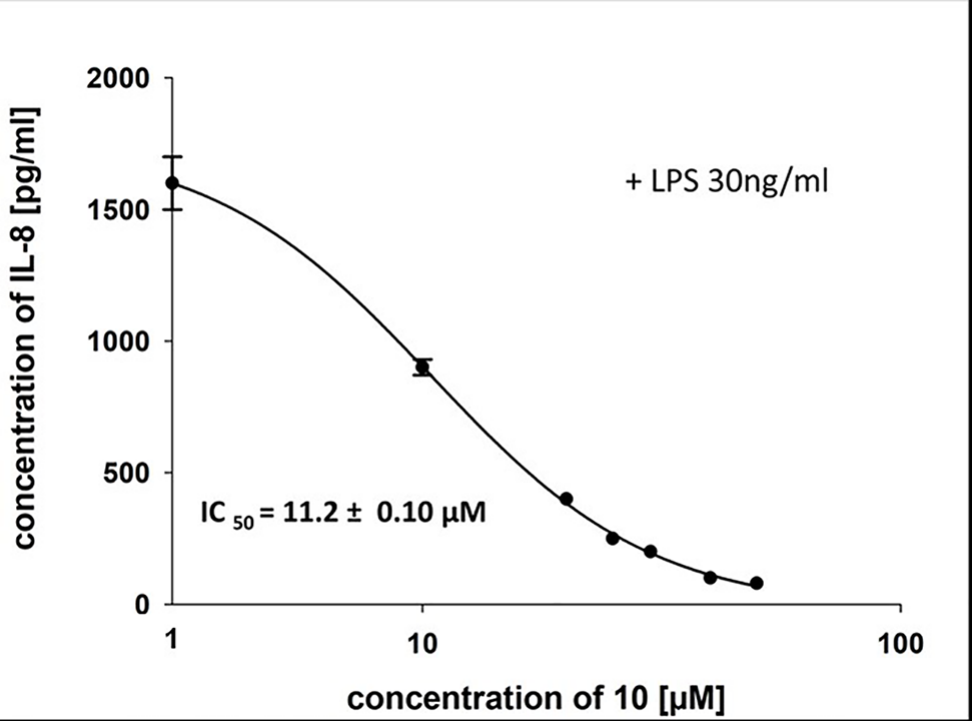
Inhibition of IL-8 secretion in HUVECtert by β-damascenone (**10**). Cells were pretreated with **10** for 15 min followed by stimulation with LPS (30 ng/ml) for 4 h. The IL-8 concentration in the medium was quantified by ELISA. Bay 11-7082 at 5 µM served as positive control. 0.1% MeOH was used as solvent control. Results are expressed as mean values ± SE.

**Figure 7:**
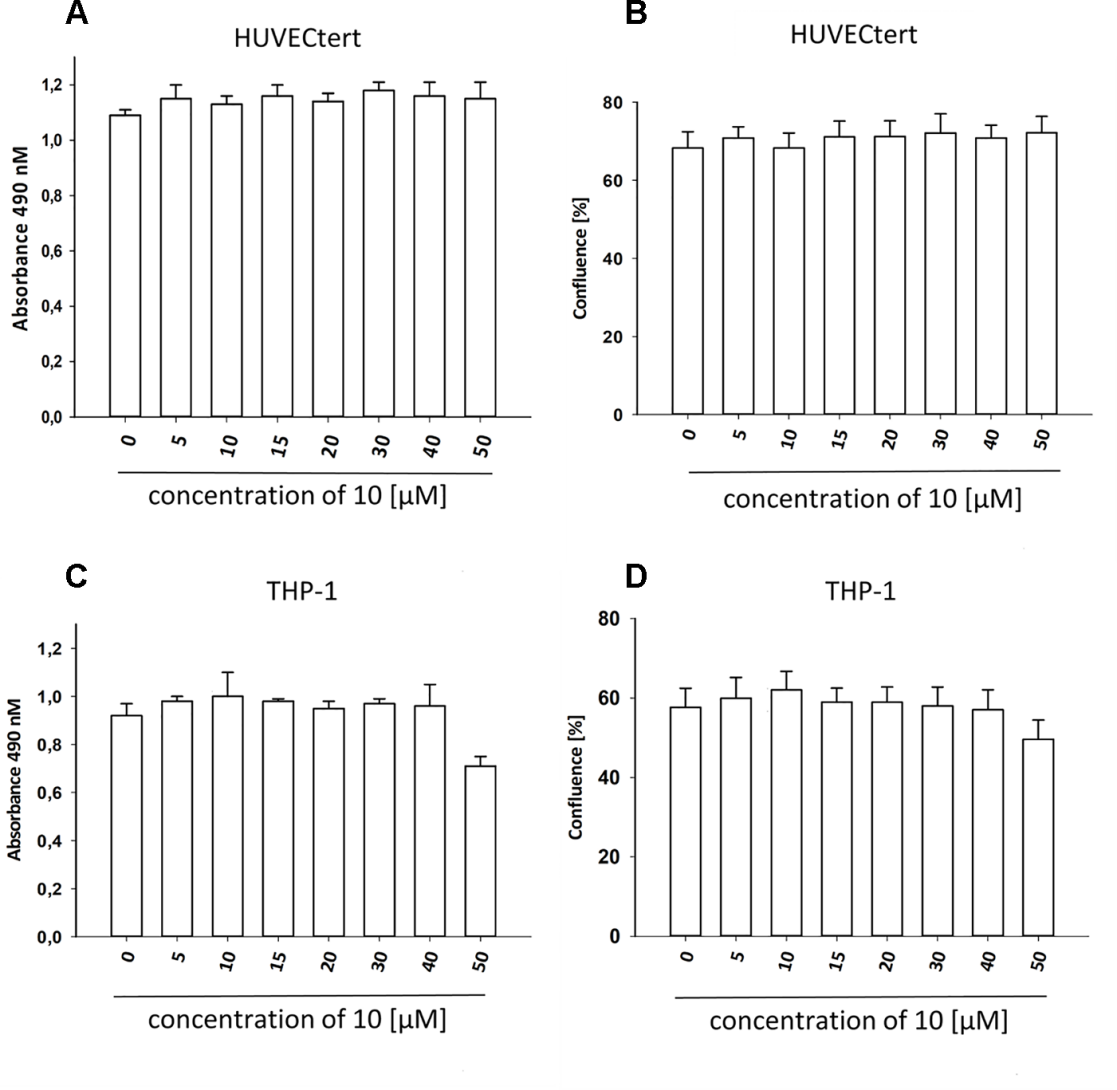
β-Damascenone at tested concentrations is not toxic. HUVECtert. **(A**, **B)** and differentiated THP-1 **(C**, **D)** cells were treated with different concentrations of β-damascenone. 0.2% DMSO served as vehicle control. After 24 h, confluence **(B**, **D)** and metabolic activity (XTT assay, **A** and **C**) were determined. The Figure presents mean values ± SD of two independent experiments with six biological replicates each.

**Figure 8:**
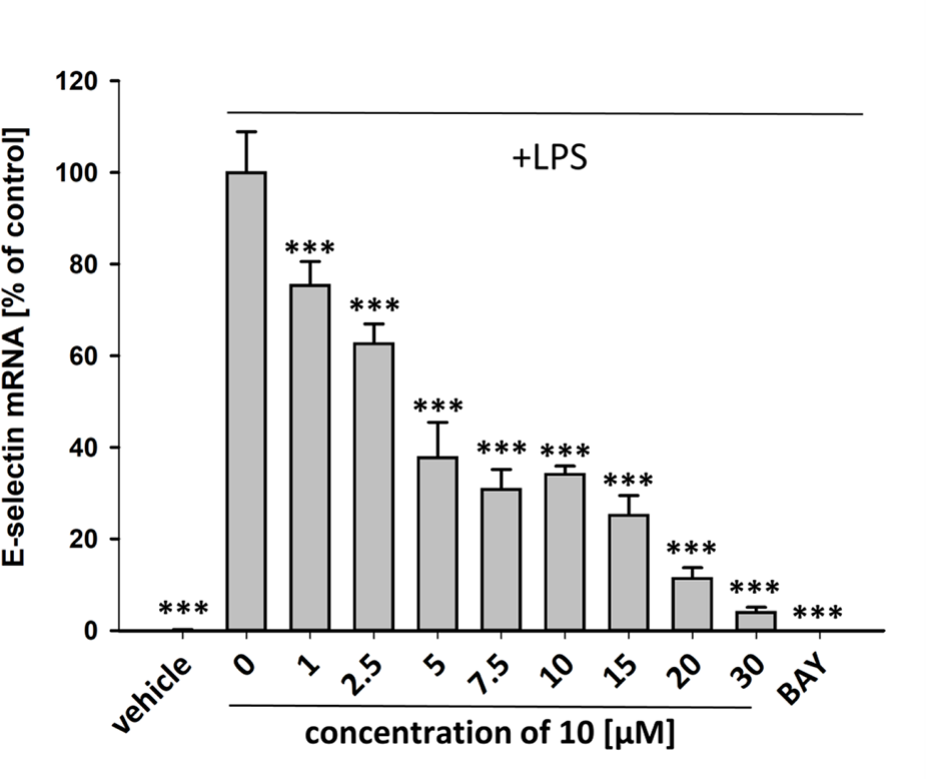
Inhibition of E-selectin mRNA expression in HUVECtert cells stimulated with LPS. Cells were pretreated with test compounds for 30 min followed by stimulation with LPS (20 ng/ml) for 4 hours. Basal values refer to vehicle-stimulated cells. Bay-11–7082 (5 µM) served as positive control. Results are normalized to β2-microglobulin. Data are presented as mean ± SE (n=4). P-values vs LPS-stimulated group are shown as ***<0.001.

**Figure 9:**
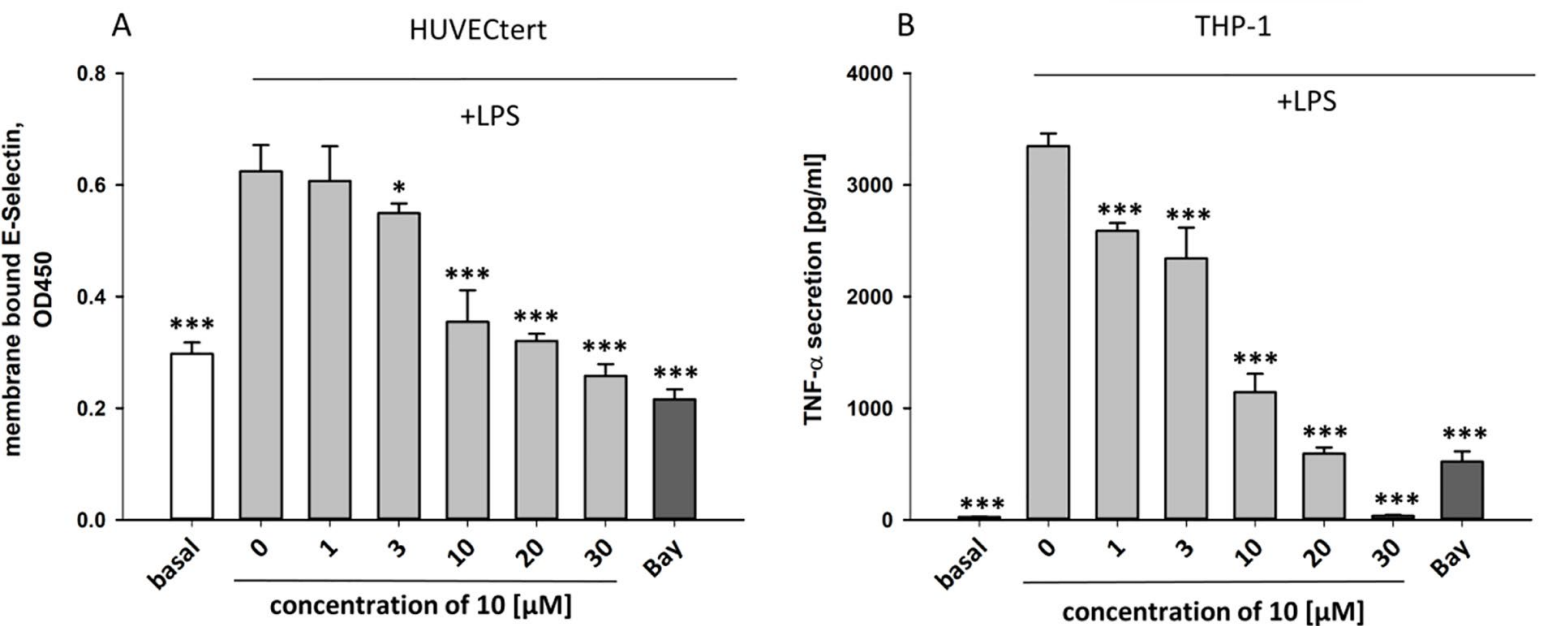
Inhibition of **(A)** protein expression of membrane bound E-selectin in HUVECtert cells and **(B)** TNF-α protein secretion in THP-1cells stimulated with LPS. Cells were pretreated with different concentrations of β-damascenone (**10**) for 30 min followed by stimulation with LPS (30 ng/ml) for 4 hours. Basal values refer to unstimulated cells. Bay-11–7082 (5 µM) served as positive control. Blank values were subtracted from readouts. Data are presented as mean ± SE (n=6). P-values vs LPS-stimulated group are shown as *< 0.5, **<0.01 ***<0.001.

To characterize the anti-inflammatory properties of **10**, endothelial cells (HUVECtert) were pretreated with this compound and then stimulated with LPS for 4 h. **10** inhibited the induction of typical NFκB-regulated pro-inflammatory genes, i.e. E-selectin, ICAM-1, and VCAM-1 (**Figures 5A–C**), as well as IL-8 (**Figure 6**). Similarly, in the monocyte-like cell line THP-1, **10** inhibited the induction of the NFκB-responsive genes TNF-α and IL-1β (**Figures 5D, E**). The effects were not due to cytotoxicity of **10**, as illustrated by the lack of cytotoxic effect after 24 h (**Figure 7**). The concentration dependence of the anti-inflammatory action of **10** was analyzed using E-selectin mRNA expression in HUVECtert cells as readout. The inhibition of E-selectin mRNA expression was concentration-dependent showing a residual activity of 37.9% at a concentration of 5 µM (**Figure 8**). Similar effects were observed for the inhibition of E-selectin protein expression on the surface of HUVECtert cells (**Figure 9**) and the secretion of TNFα protein by LPS-stimulated THP-1 cells (**Figure 9**). The data of **Figure 9** show that **10** not only reduced induction of mRNA of pro-inflammatory genes, but also blocked their expression at the protein level.

**Figure 10:**
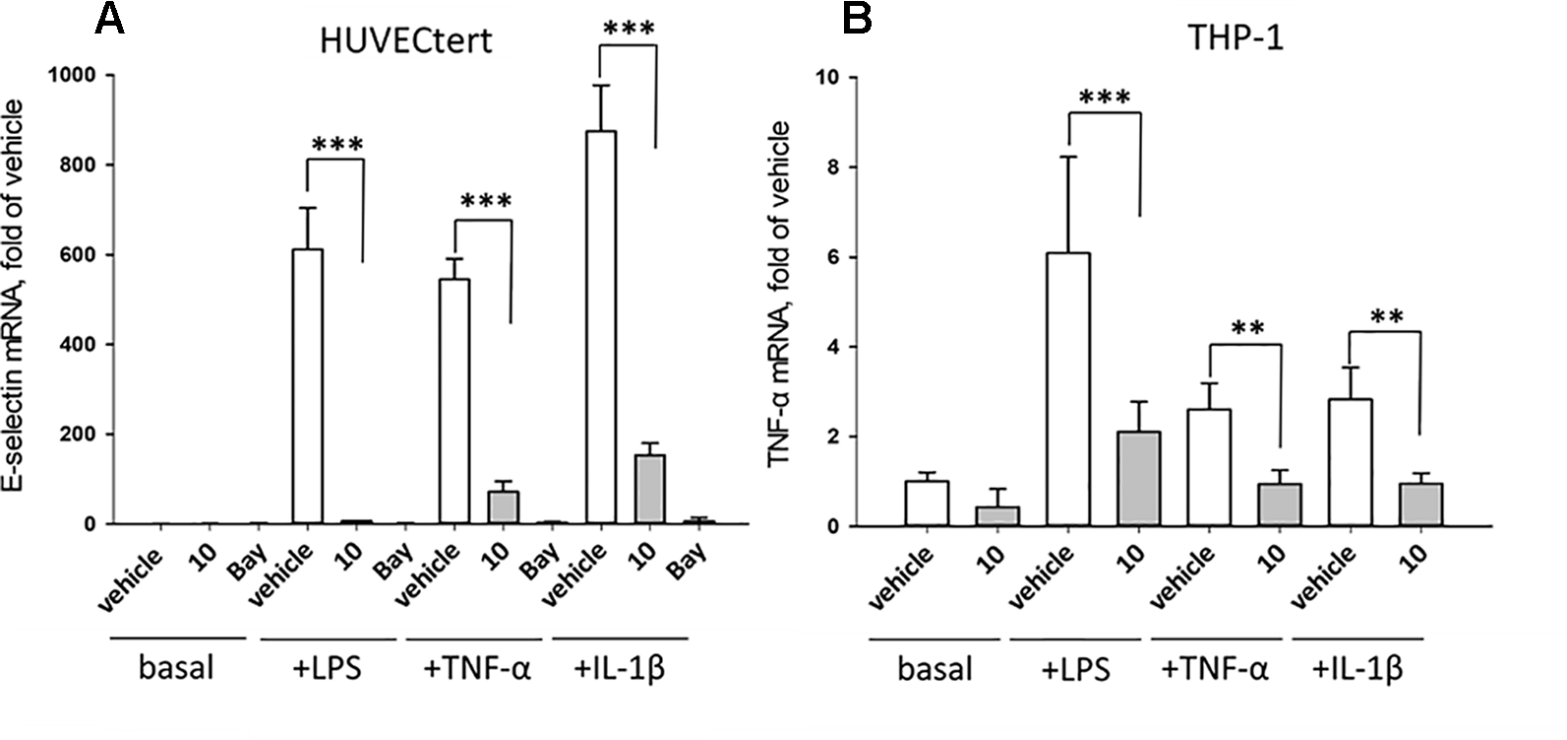
Inhibition of **(A)** E-selectin mRNA expression in HUVECtert and **(B)** TNF-α mRNA expression in THP-1 cells stimulated with different agonists of NF-κB pathway. Cells were pretreated with β-damascenone (**10**) for 30 min followed by stimulation with LPS (30 ng/ml), TNF-α (0.3 ng/ml) or IL-1β (1 ng/ml) for 4 hours. Basal values refer to vehicle-stimulated cells. Isolation of total RNA, cDNA synthesis and real-time PCR were performed as described in materials and methods section. Results are normalized to β2-microglobulin. Data are presented as mean ± SE (n=4). P-Values are shown as **<0.01 ***<0.001

The experiments described in the previous paragraph were performed using cells stimulated by bacterial LPS. Further experiments analyzed whether **10** can also inhibit effects induced by pro-inflammatory cytokines that bind to different receptors than LPS. To this end, endothelial cells were stimulated with TNF-α, IL-1β, or LPS in the absence or presence of **10**. All stimuli upregulated E-selectin mRNA in HUVECtert, and the induction was significantly inhibited by **10** (**Figure 10**). Similarly, **10** inhibited the induction of TNF-α mRNA in THP-1 cells treated by all three pro-inflammatory stimuli (**Figure 10**). Since these stimuli activate different receptors with different downstream signaling steps, we hypothesized that **10** inhibits the transduction of inflammatory signals at a post-receptor level. The signaling pathway, in which all three upstream pathways converge, is the NF-κB signaling pathway (Sun, 2017).

**Figure 11:**
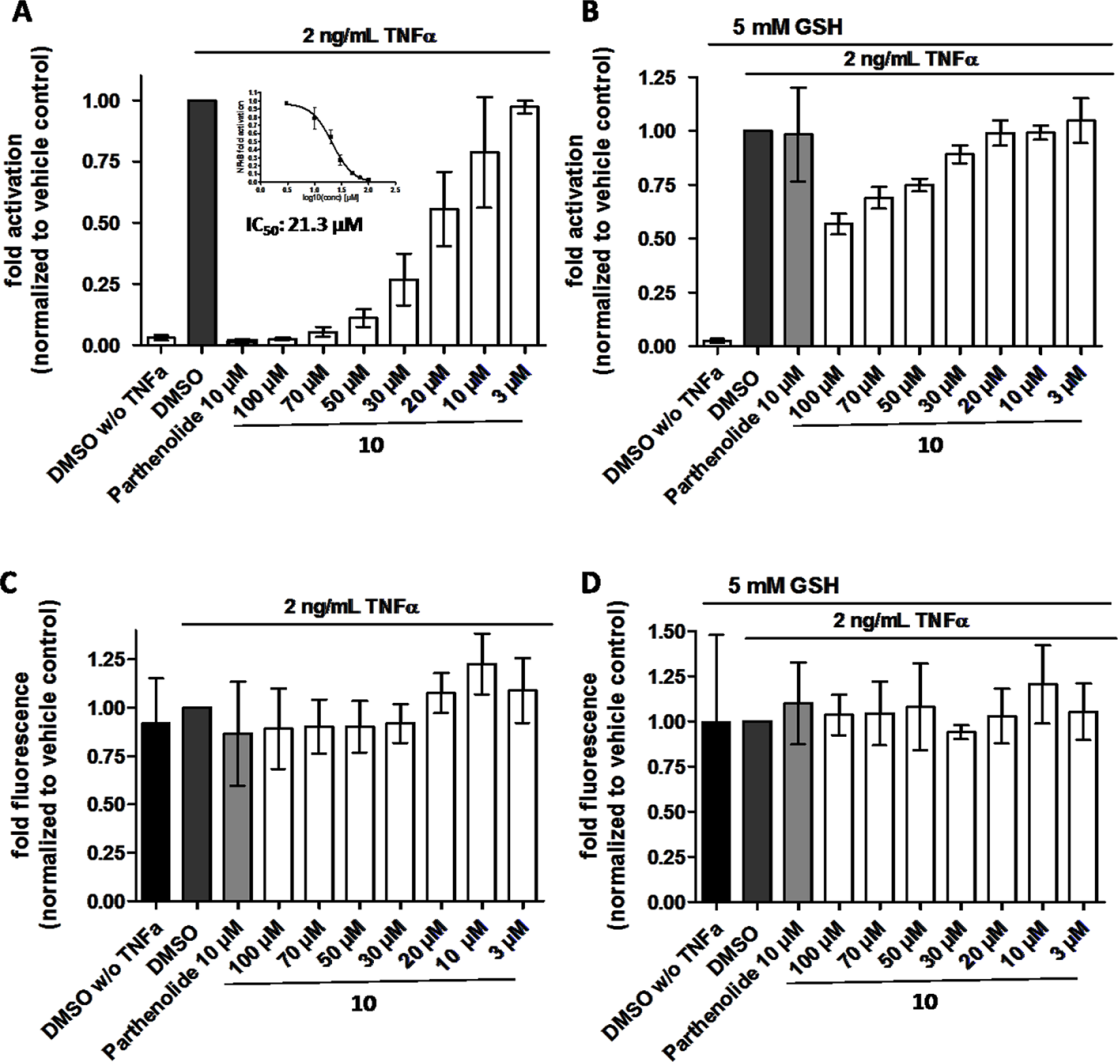
*β-*Damascenone (**10**) inhibits TNF-α (2 ng/ml)-induced NF-κB-driven luciferase reporter gene transactivation concentration dependently; the inhibition is reversed in the presence of 5 mM glutathione (GSH). HEK293/NF-κB-luc cells were loaded with cell tracker green (CTG), a probe for vital staining. After 24 hours cells were pretreated for 1 hour with the indicated compounds, and in **(B)** in addition with 5 mM GSH (as indicated), and activated with TNF-α (2 ng/ml) for 4 hours. Then, **(A** and **B)** luciferase activity and **(C** and **D)** CTG fluorescence were measured. Luciferase activity is shown normalized to CTG fluorescence. Parthenolide (10 µM) served as positive control. Data are presented as mean ± SD (n = 4) and are normalized to the vehicle control DMSO.

In order to directly test whether the anti-inflammatory activity of **10** may result from inhibiting the NF-κB signaling pathway, HEK 293 cells stably transfected with a NF-κB-driven luciferase gene were used. **10** inhibited the NF-κB-driven reporter gene transactivation concentration-dependently (**Figure 11A**). The IC_50_ value obtained was 21.3 µM. The effect was not due to cytotoxicity of **10** as documented by the Cell Tracker Green fluorescence (**Figure 11C**). **10** is an active electrophile, and in contrast to other megastigmane aglycones, **10** possesses two active sites. This characteristic feature makes **10** a reactive Michael acceptor able to react with thiol groups found in many signal-transducing proteins (Gerhauser et al., 2009). We, therefore, tested whether the inhibitory effect of **10** can be inhibited by adding glutathione acting as decoy for reactive Michael acceptors (Heilmann et al., 2001). **Figure 11B** shows that 5 mM glutathione largely blocked the NF-κB inhibitory activity of **10** as well as that of the positive control parthenolide, which is also a reactive Michael acceptor (Kwok et al., 2001).

Electrophiles have been shown to covalently modify IKKβ, thus leading to its inactivation (Kapahi et al., 2000; Kwok et al., 2001). This kinase phosphorylates the NF-κB inhibitor IκB and targets it for proteasomal degradation, finally leading to NF-κB activation. Thus, inactivation of IKKβ by electrophiles results in inhibition of NF-κB independently of the upstream mechanisms that induced activation of IKKβ.

In summary, the major finding of this investigation is that β-damascenone is a major active compound of *Epipremnum pinnatum*, and that it inhibits NF-κB signaling pathway *in vitro* in human cellular systems that had been activated with different inflammatory stimuli, and that this is most likely mediated by the electrophilic property of the compound. Our results are in agreement with the data of Gerhauser et al., 2009), who showed that **10** was able to inhibit iNOS expression in LPS-stimulated murine macrophages and to activate the transcription factor Nrf2. Also, the activation of Nrf2 can be promoted by an electrophilic insult that covalently modifies Keap1, which finally leads to Nrf2 accumulation, nuclear translocation and transcriptional activation of respective target genes (Matzinger et al., 2018). Thus, **10** as an electrophilic compound can simultaneously activate Nrf2 and inhibit NF-κB, finally resulting in an antioxidant defense and suppression of pro-inflammatory target genes. Such a pharmacodynamic profile may be especially beneficial for treatment of acute inflammatory conditions, e.g., ischemia-reperfusion injury, which is usually accompanied by severe oxidative stress. Therefore, our results show that megastigmane derivatives, in particular β-damascenone and its precursors, may be responsible for the anti-inflammatory effects of *Epipremnum pinnatum via* inhibition of the NF-κB pathway. It is most likely that its α,β-unsaturated carbonyl moiety is responsible for this effect. Since α,β-unsaturated carbonyl moieties interact with many signal-transduction proteins, it needs to be clarified whether β-damascenone expresses its effects only by targeting proteins within the NF-κB and Nrf2 signaling cascade, or whether it also affects other signaling pathways.

## Author Contributions

Bio-activity guided isolation was performed by S-PP. Structure elucidation including LC-MS and GC-MS was performed by S-PP and OK. S-PP performed the COX-2 mRNA assay. Analysis of NF-κB dependent genes, TNF-α and E-selectin ELISA was performed by TP. NK and TP did cell viability assays. SH, SL, and JR performed the Luciferase reporter gene assays. Graphs and statistical analysis were made by contributors who performed the experiments. S-PP wrote the original draft of the manuscript. RB, VB, VD, and TP designed and conceived the study, contributed to writing and revisions of the manuscript. All authors contributed to writing and revisions of the manuscript.

## Funding

The work was partially supported by a grant from the Austrian Science Fund (P 27682-B30 to VB).

## Conflict of Interest

The authors declare that the research was conducted in the absence of any commercial or financial relationships that could be construed as a potential conflict of interest.
