# Supplementary material for: C13 Megastigmane Derivatives From Epipremnum pinnatum: β-Damascenone Inhibits the Expression of Pro-Inflammatory Cytokines and Leukocyte Adhesion Molecules as Well as NF-κB Signaling
Source: Front Pharmacol. 2019 Nov 28;10:1351. doi: 10.3389/fphar.2019.01351 (PMC6892967; doi:10.3389/fphar.2019.01351)
Supplement: Supplementary file 1 [file DataSheet_1.docx]

**C13 Megastigmane Derivatives from Epipremnum pinnatum ̶
β-Damascenone Inhibits the Expression of Pro-inflammatory Cytokines and Leukocyte Adhesion Molecules as well as NF-κB signaling**

**San-Po Pan^1^, Teresa Pirker^1^, Olaf Kunert^2^, Nadine Kretschmer^1^, Scarlet Hummelbrunner^3^, Simone L. Latkolik^3^, Julia Rappai^3^, Verena Dirsch^3^, Valery Bochkov^2^, Rudolf Bauer*^1^**

^1^Institute of Pharmaceutical Sciences, Department of Pharmacognosy, University of Graz, Graz, Austria

^2^Institute of Pharmaceutical Sciences, Department of Pharmaceutical Chemistry, University of Graz, Graz, Austria

^3^Department of Pharmacognosy, Molecular Targets, University of Vienna, Vienna, Austria

*** Correspondence:**Rudolf Bauer
rudolf.bauer@uni-graz.at

**Supplementary data**

**List of Figures and tables**

Figure S1.  ^1^H spectrum of **1**

Figure S2. HSQC spectrum of **1**

Figure S3. ^1^H NMR spectrum of **2** and **3**

Figure S4. HSQC spectrum of compound **2** and **3**

Figure S5. GC-MS chromatogram of identified megastigmane aglycones **10-13**

Figure S6. EI Mass spectrum (GC-MS) of β-damascenone **(10)** compared to library data

Figure S7. EI Mass spectrum (GC-MS) of megastigmatrienone **(11)** compared to library data

Figure S8. EI Mass spectrum (GC-MS) of 3-Oxo-7,8-dihydro-alpha-ionol **(12)** compared to library data

Figure S9. EI Mass spectrum (GC-MS) of 3-Hydroxy-beta-damascone **(13)** compared to library data

Table S1. NMR Spectroscopic Data [methanol-d_4_, 599.85 MHz (^1^H) and 150.84 MHz (^13^C), δ (ppm)] of **1 - 3**

**
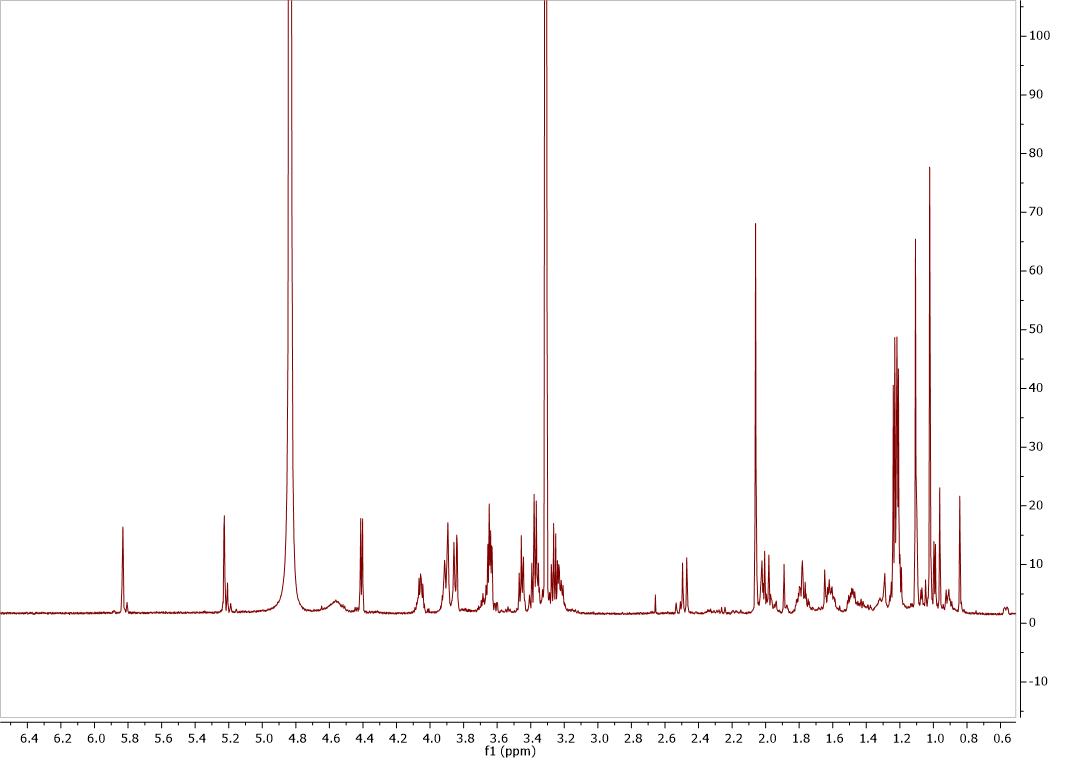
**

**Figure 1.** ^1^H spectrum of **1**

**
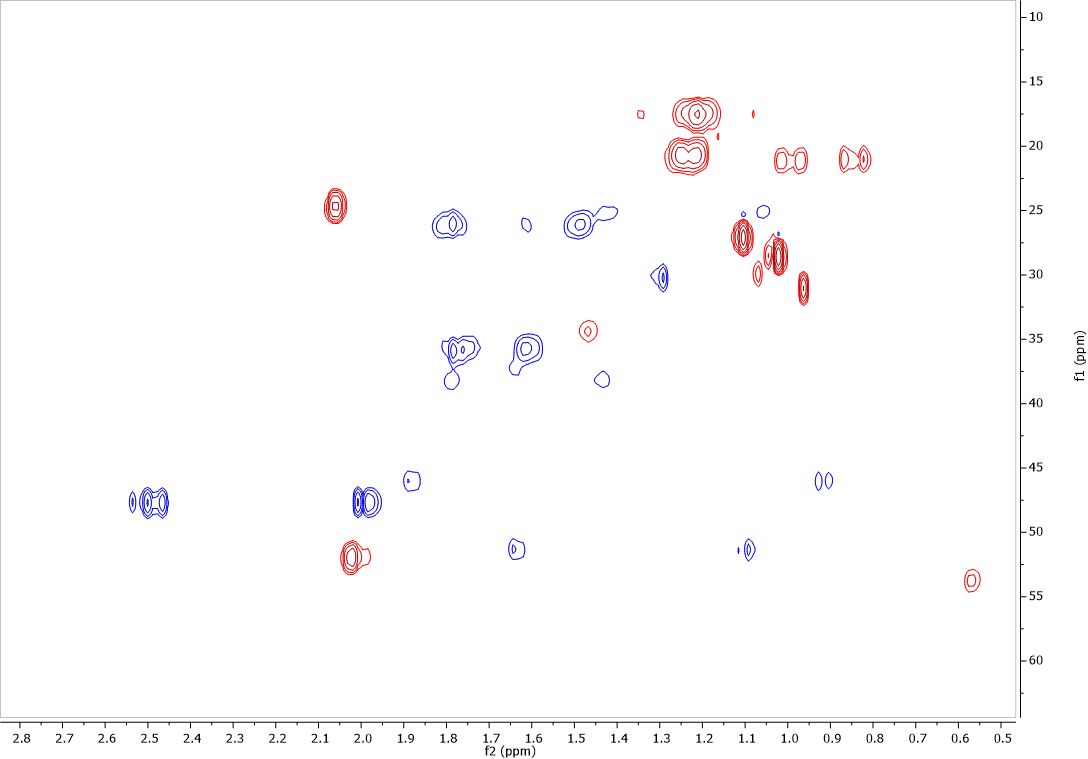
**

**Figure 2.** HSQC spectrum of **1**

**
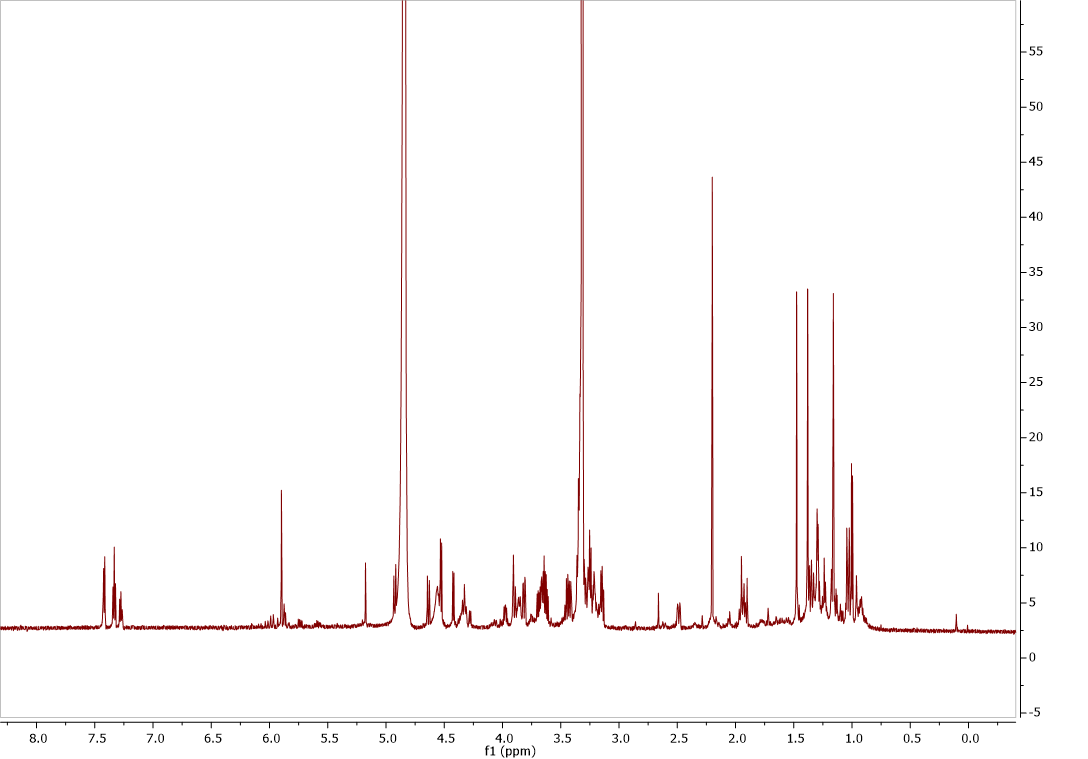
**

**Figure 3.** ^1^H NMR spectrum of **2** and **3**

**
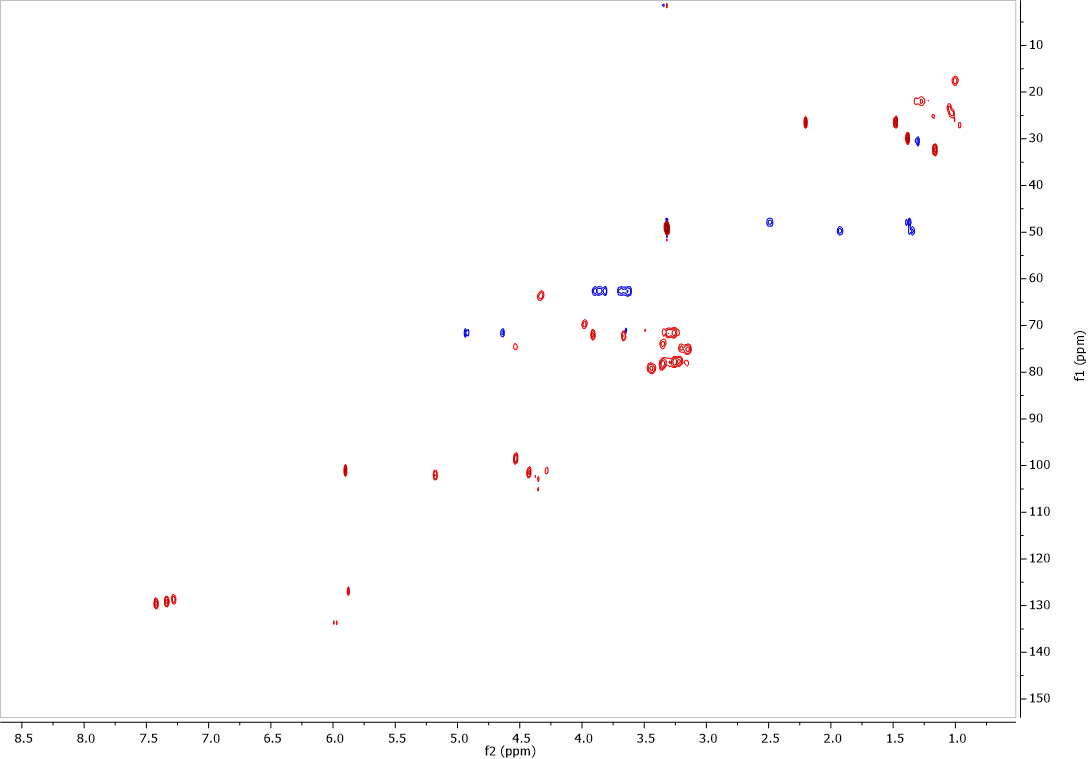
**

**Figure 4.** HSQC spectrum of **2** and **3**


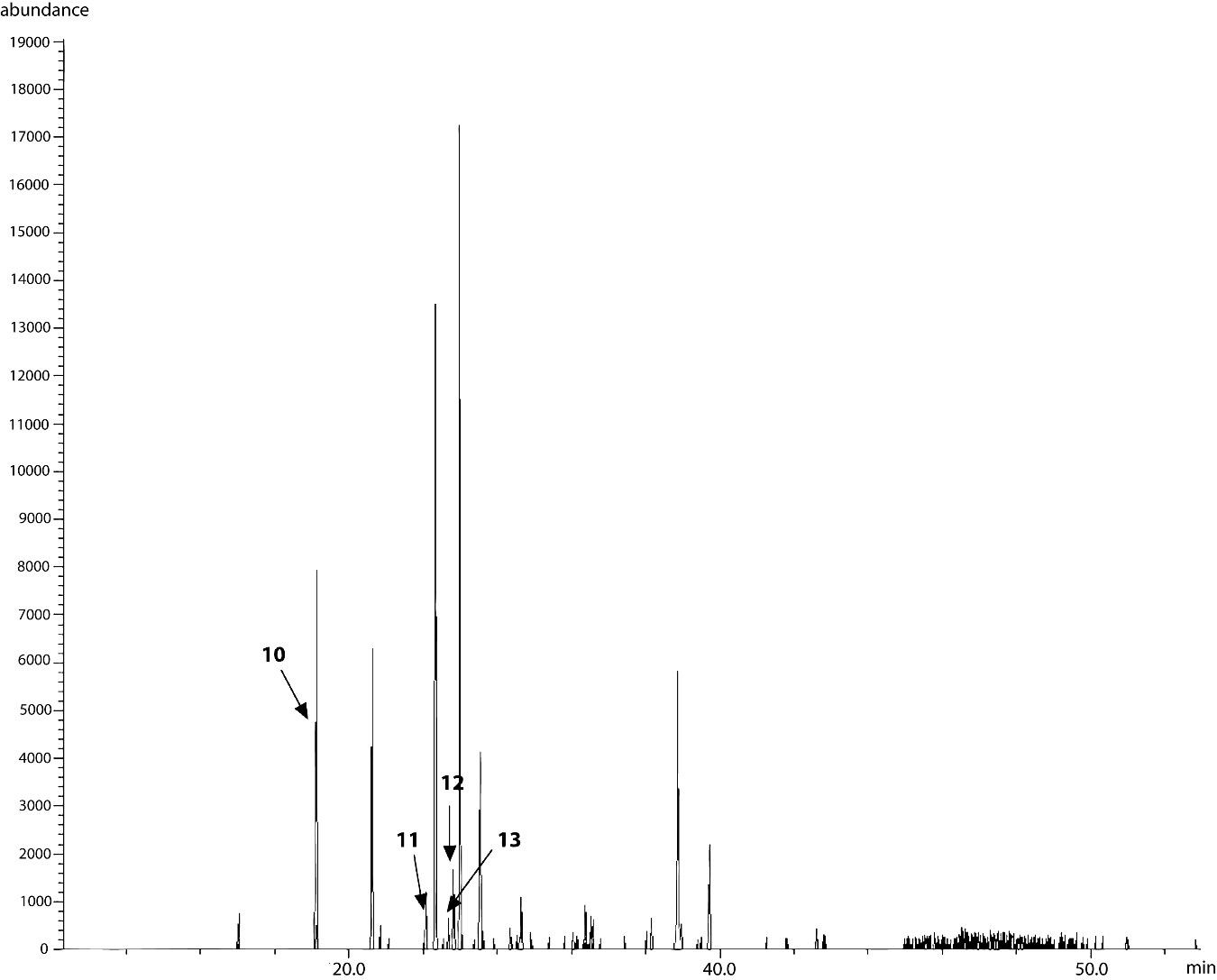


**Figure S5**. GC-MS chromatogram of identified megastigmane aglycones **10-13**.


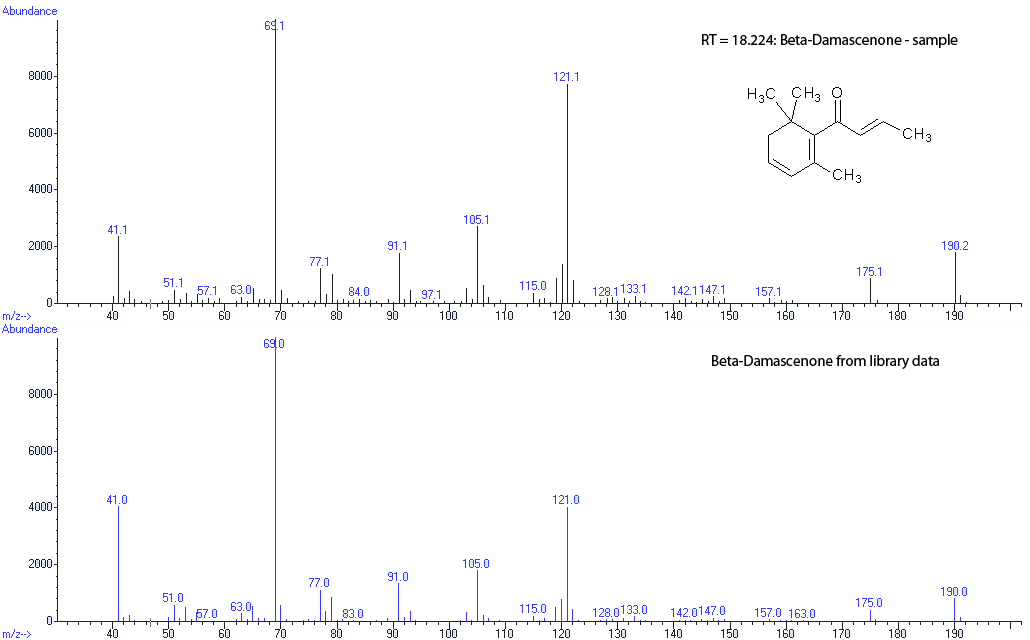


**Figure S6.** EI Mass spectrum (GC-MS) of β-damascenone **(10)** compared to library data


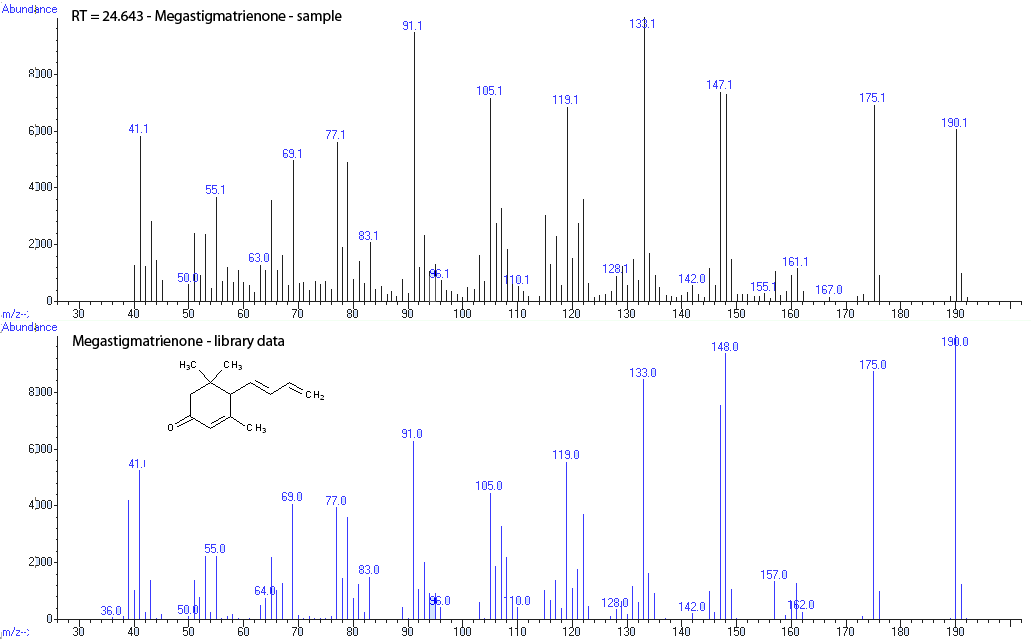


**Figure S7.** EI Mass spectrum (GC-MS) of megastigmatrienone **(11)** compared to library data


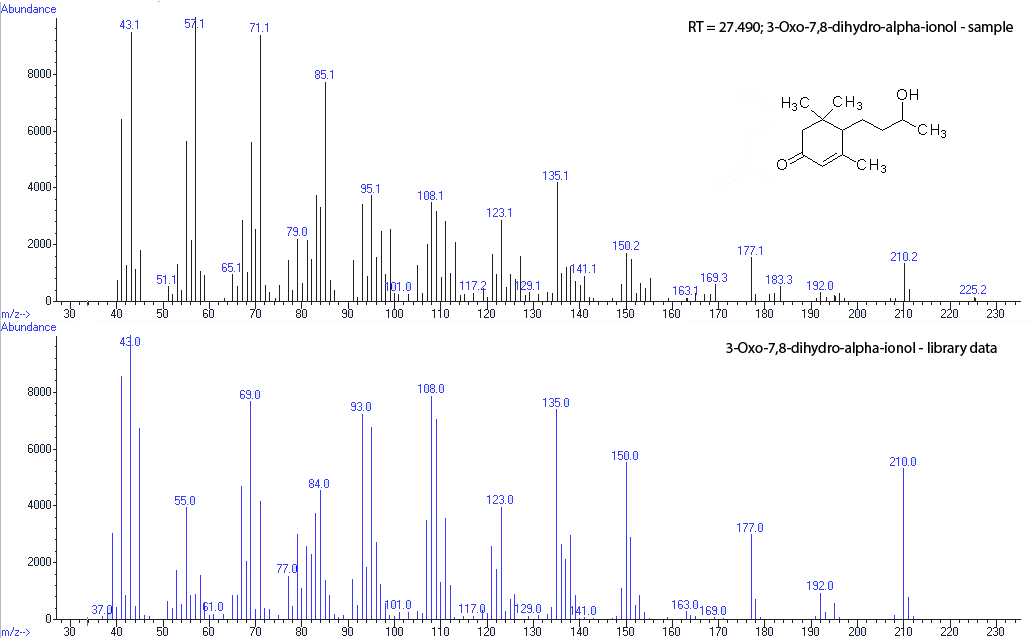


**Figure S8.** EI Mass spectrum (GC-MS) of 3-Oxo-7,8-dihydro-alpha-ionol **(12)** compared to library data


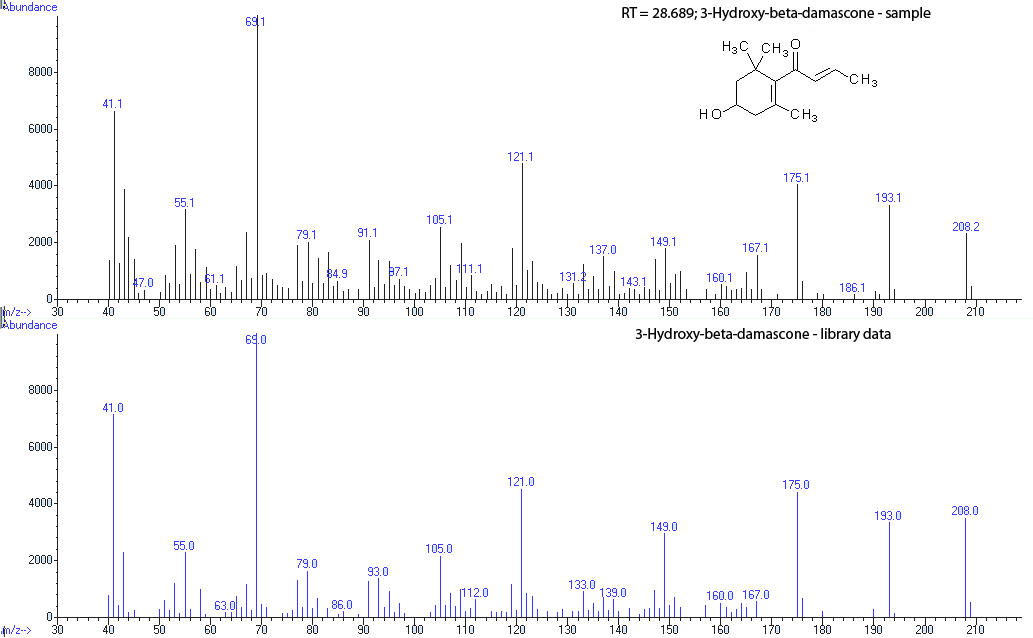


**Figure S9.** EI Mass spectrum (GC-MS) of 3-Hydroxy-beta-damascone **(13)** compared to library data

**Table S1.** NMR spectroscopic data [methanol-d_4_, 599.85 MHz (^1^H) and 150.84 MHz (^13^C), δ (ppm)] of **1 - 3**

|  | **Gusanlungionoside C**  **(1)** | | **Phenylmethyl-2-O-(6-O-rhamnosyl)-ß-D-galactopyranoside (2)** | | **Citroside A**  **(3)** | |
| --- | --- | --- | --- | --- | --- | --- |
| Position | δ_C_ | δ_H_ (J in Hz) | δ_C_ | δ_H_ (J in Hz) | δ_C_ | δ_H_ (J in Hz) |
| 1 | 37.0, C | - | 138.4, C | - | 37.0, C | - |
| 2 | 47.6, CH_2_ | 2.50, 1.98 d (16 Hz) | 129.7, C | 7.42 (d; 7.6 Hz) | 49.7, C | 1.95, 1.36 |
| 3 | 202.0, C | - | 129.0, C | 7.33 (t; 7.5 Hz) | 63.5, C | 4.35 |
| 4 | 125.0, CH | 5.83 s | 128.7, C | 7.28 (t; 7.5 Hz) | 47.9, CH_2_ | 2.50, 1.41 |
| 5 | 169.6, C | - | 129.0, C | 7.33 (t; 7.5 Hz) | 78.4, C | - |
| 6 | 52.1, C | 2.02 m | 129.7, C | 7.42 (d; 7.6 Hz) | 118.9, C | - |
| 7 | 26.1, CH_2_ | 1.80, 1.48 m | 71.5, CH_2_ | 4.64 (d; 11.3 Hz)  4.94 (d; 11.3 Hz) | n. d. | - |
| 8 | 35.9, CH_2_ | 1.78, 1.62 m |  |  | 101.1, CH | 5.90 s |
| 9 | 75.1, CH | 3.90 m |  |  | 200.4, C | - |
| 10 | 20.7, CH_3_ | 1.23 d |  |  | 26.6, CH_3_ | 2.20 s |
| 11 | - | - |  |  | 32.4, CH_3_ | 1.16 s |
| 12 | - | - |  |  | 29.9, CH_3_ | 1.40 s |
| 13 | 24.8, CH_3_ | 2.06 s |  |  | 26.4, CH_3_ | 1.48 s |
| 1´ | 100.5, CH | 4.40 d (7.8 Hz) | 101.6, CH | 4.42 (d; 7.2 Hz) | 98.6, CH | 4.53 (d; 7.7 Hz) |
| 2´ | 78.3, CH | 3.37 m | 79.2, CH | 3.44 m | 75.1, CH | 3.15 |
| 3´ | 78.9, CH | 3.45 t | 77.6, CH | 3.29 m | 78.3, CH | 3.36 |
| 4´ | 71.6, CH | 3.26 m | 71.4, CH | 3.28 m | 71.6, CH | 3.26 |
| 5´ | 77.3, CH | 3.23 m | n. d. | n. d. | overlapped |  |
| 6´ | 62.2, CH_2_ | 3.84, 3.65 m | 62.6, CH_2_ | 3.87, 3.68 m | overlapped |  |
| 1´´ | 101.4, CH | 5.22 d (1.4 Hz) | 102.1, CH | 5.20 (s; brs) |  |  |
| 2´´ | 71.7, CH | 3.90 m | 72.0, CH | 3.91 m |  |  |
| 3´´ | 72.0, CH | 3.63 m | 72.2, CH | 3.68 m |  |  |
| 4´´ | 73.5, CH | 3.38 m | 73.9, CH | 3.35 m |  |  |
| 5´´ | 69.5, CH | 4.06 m | 69.6, CH | 3.98 m |  |  |
| 6´´ | 17.5, CH_3_ | 1.21 d (6 Hz) | 17.6, CH_3_ | 1.00 (d; 6.6 Hz) |  |  |
